# Super‐Resolution Goes Viral: T4 Virus Particles as Versatile 3D‐Bio‐NanoRulers

**DOI:** 10.1002/adma.202403365

**Published:** 2025-01-16

**Authors:** José Ignacio Gallea, Oleksii Nevskyi, Zuzanna Kaźmierczak, Ivan Gligonov, Tao Chen, Paulina Miernikiewicz, Anna M. Chizhik, Lenny Reinkensmeier, Krystyna Dąbrowska, Mark Bates, Jörg Enderlein

**Affiliations:** ^1^ Third Institute of Physics – Biophysics Georg August University Friedrich‐Hund Platz 1 37077 Göttingen Germany; ^2^ Hirszfeld Institute of Immunology and Experimental Therapy Polish Academy of Sciences Rudolfa Weigla 12 Wroclaw 53–114 Poland; ^3^ Research and Development Centre Regional Specialist Hospital Kamienskiego 73a Wroclaw 53–114 Poland; ^4^ Lenny Reinkensmeier Department of Optical Nanoscopy Institute for Nanophotonics Hans‐Adolf‐Krebs‐Weg 1 37077 Göttingen Germany; ^5^ Faculty of Medicine Department of Preclinical Sciences Pharmacology and Medical Diagnostics Wrocław University of Science and Technology Hoene‐Wrońskiego 13 c Wrocław 58–376 Poland; ^6^ Cluster of Excellence “Multiscale Bioimaging: from Molecular Machines to Networks of Excitable Cells” (MBExC) Universitätsmedizin Göttingen Robert‐Koch‐Str. 40 37075 Göttingen Germany

**Keywords:** bacteriophages, DNA‐PAINT, nanorulers, super‐resolution fluorescence microscopy, virus structure

## Abstract

In the burgeoning field of super‐resolution fluorescence microscopy, significant efforts are being dedicated to expanding its applications into the 3D domain. Various methodologies have been developed that enable isotropic resolution at the nanometer scale, facilitating the visualization of 3D subcellular structures with unprecedented clarity. Central to this progress is the need for reliable 3D structures that are biologically compatible for validating resolution capabilities. Choosing the optimal standard poses a considerable challenge, necessitating, among other attributes, precisely defined geometry and the capability for specific labeling at sub‐diffraction‐limit distances. In this context, the use of the non‐human‐infecting virus, bacteriophage T4 is introduced as an effective and straightforward bio‐ruler for 3D super‐resolution imaging. Employing DNA point accumulation for imaging in nanoscale topography (DNA‐PAINT) along with the technique of astigmatic imaging, the icosahedral capsid of the bacteriophage T4, measuring 120 nm in length and 86 nm in width, and its hollow viral tail is uncovered. This level of detail in light microscopy represents a significant advancement in T4 imaging. A simple protocol for the production and preparation of samples is further outlined. Moreover, the extensive potential of bacteriophage T4 as a multifaceted 3D bio‐ruler, proposing its application as a novel benchmark for 3D super‐resolution imaging in biological studies is explored.

## Graphical abstract

Various super‐resolution fluorescence microscopy methods achieve nanoscale resolution, vital for visualizing subcellular structures. Choosing suitable biological standards is challenging, demanding precise geometry and specific labeling capabilities. Utilizing T4 bacteriophage as a 3D‐Bio‐NanoRuler is proposed. With DNA‐PAINT and astigmatic imaging, detailed viral structures are revealed, offering a simple sample protocol and suggesting T4's potential as a benchmark in microscopy studies.

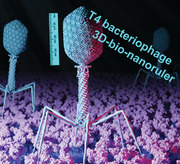

## 1 Introduction

The advent of super‐resolution fluorescence microscopy techniques, breaking through the diffraction limit of light, has revolutionized our ability to visualize life at its most fundamental level. Among these, Single Molecule Localization Microscopy (SMLM) methods stand out for their capacity to delineate biological structures, such as cellular organelles and protein complexes, with near‐molecular resolution.^[^ ^1^, ^2^ ^]^ These techniques have illuminated the once‐obscure realm of “sub‐microscopic” infectious agents, namely viruses, which conventional light microscopy struggled to detail.^[^ ^3^ ^]^ Initially confined to 2D imaging, these pioneering technologies have expanded into the 3D sphere, enhancing our understanding of biological specimens' true complexities.^[^ ^4^, ^5^, ^6^, ^7^ ^]^ Recent advancements have achieved isotropic resolution within the nanometer range, enabling the precise visualization of 3D structures.^[^ ^8^, ^9^, ^10^, ^11^, ^12^ ^]^

While SMLM techniques have gained acceptance in biological research, they still pose challenges, particularly with respect to the quantification and analysis of 3D SMLM images. Consequently, tools and methods that validate the accuracy and reliability of these techniques are essential. Scientists have employed various strategies to tackle this, including image analysis and experimental methods. A common approach involves measuring localization precision, which indicates the uncertainty in determining individual molecules' positions.^[^ ^13^ ^]^ However, this can overestimate resolution capabilities by not accounting for factors such as label density and sample drift. Other methods, like Fourier Ring Correlation (FRC)^[^ ^14^ ^]^ and decorrelation analysis,^[^ ^15^ ^]^ offer direct resolution estimates from super‐resolved images but may miss local variations and depend heavily on proper image capture and processing.

To bridge these gaps, researchers have turned to reference structures, often called “standards” or “rulers.” These structures ideally possess a well‐defined geometric arrangement, have multiple reference points strategically placed at distances below the diffraction limit, allow for common tagging schemes, and are stable and highly reproducible. The use of these rulers allows for correlating the estimated positions of emitters to their true position established by the reference points, thereby checking the localization accuracy.^[^ ^13^ ^]^ They also facilitate the estimation of spatial resolution by analyzing the distances between fluorophores positioned at known intervals, effectively acting as molecular rulers.

DNA origami structures, with their customizable shapes and precise fluorophore placement, exemplify such standards.^[^ ^16^, ^17^ ^]^ These structures are long single‐stranded DNAs folded by using numerous short DNA “staple” strands to form a well‐defined 2D or 3D structure.^[^ ^16^ ^]^ DNA origami, due to its designable nature, provides exceptional structural versatility and enables the accurate positioning of fluorophores at specific sites across desired length scales. By contrast, as DNA origami is in vitro assembled, nucleic acid‐based structures, may not be optimal for genetic tagging with proteinaceous tags such as fluorescent proteins or self‐labeling enzymes, and these structures are predominantly suited for cell‐free environments.^[^ ^18^ ^]^ Other notable examples involve employing cellular structures such as microtubules or the nuclear pore complex (NPC) as reference standards. Microtubules, in particular, are frequently utilized to evaluate spatial resolution through the measurement of their cross‐sectional fluorescence profile. This method, which was also performed in vitro,^[^ ^19^ ^]^ facilitates the determination of peak‐to‐peak distances between fluorophores positioned on opposing sides of the microtubule's hollow cylindrical structure. Nonetheless, this technique is constrained by the inherent width of the microtubules (≈25 nm, excluding linkage error) and its relatively simple structure. A second example of a biological calibration structure is NPCs, which have recently been demonstrated as a reference standard.^[^ ^18^ ^]^ These cylindrical protein complexes, situated in the nuclear envelope, offer an important resource for resolution assessment. For instance, labeling NUP96, a component protein of NPCs, enables distance measurements ranging from ≈12 to 107 nm under optimal conditions. This allows for direct evaluation of whether the resolution exceeds these distances. However, the application of NPCs is confined to cellular imaging contexts.

In a recent study, Helmrich et al. introduce the proliferating cell nuclear antigen (PCNA) as a protein‐based nanoruler capable of achieving sub‐10 nm resolution.^[^ ^20^ ^]^ This homo‐trimeric protein, essential for DNA replication and repair and measuring 8 nm, has undergone genetic code expansion to include three labeling sites strategically spaced at 6 nm intervals. This modification enables precise labeling through biorthogonal click chemistry. Moreover, PCNA demonstrates remarkable stability within cellular environments, offering benefits in scenarios where accurate stoichiometry is crucial. However, this nanoruler has some limitations, including its inability to measure in three dimensions, limited length versatility, and lack of multitarget potential.

In this work, we introduce the novel capability of the non‐pathogenic virus, bacteriophage T4, to act as a versatile 3D‐Bio‐NanoRuler, which may simplify the calibration and quantification of super‐resolution fluorescence microscopy studies. This naturally occurring DNA‐protein complex, with its icosahedral capsid measuring 120 nm in length and 86 nm in width, paired with a cylindrical hollow viral tail, forms a unique 260 nm geometric rocket‐shaped structure. By employing the DNA point accumulation for imaging in nanoscale topography (DNA‐PAINT),^[^ ^21^, ^22^ ^]^ in tandem with optical astigmatism,^[^ ^23^ ^]^ we have achieved detailed visualization of T4's 3D structure with an unparalleled level of clarity using light microscopy. Additionally, we demonstrate T4's utility in assessing microscope performance, where the phage offers an extremely rigid 3D structure for calibrating microscopes with nanometer accuracy. Our streamlined preparation method not only yields sufficient virus for ≈400 SMLM measurements, with a density of ≈500 virus particles per 400 µm^2^ but also ensures that the majority of viruses are oriented perpendicular to the coverslip, making them promising for use as 3D standards. Finally, we investigate their use as universal nanometer‐scale rulers, highlighting their potential for facilitating super‐resolution imaging for biological research.

## 2 Results

### 2.1 3D‐Bio‐NanoRuler Sample Preparation and 3D Imaging

T4 are double‐stranded DNA viruses of the *Straboviridae* family within *Caudoviricetes*, that infect *Escherichia coli* (E. coli) bacteria. Morphologically, T4 represents myoviruses, having an icosahedral head and a long, contractile but non‐flexible tail. These viruses are ubiquitous in nature and can be safely manipulated in laboratories operating at the lowest biosafety level (BSL‐1). This safety profile contributes to the widespread use of T4 as a model organism for biological research and as a standard tool for assessing aerosol containment in cell sorters.^[^ ^24^ ^]^ T4 viruses are readily available on the market and can be replicated and purified using several established simple protocols, two of which we have used and are described in Section 4.

**Figure 1:**
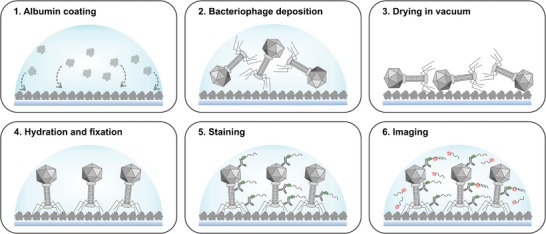
Preparation scheme of the T4 bacteriophage sample for DNA‐PAINT imaging.

Another fascinating feature of T4 is its ability to orient itself perpendicularly to an albumin‐coated substrate, a phenomenon that, to our knowledge, has not been previously documented. We discovered this through the use of an easy sample preparation protocol outlined in **Figure** **1** and detailed in Section 4. This protocol consists of five main preparation steps for imaging: 1‐ coating the coverslip with bovine serum albumin (BSA); 2‐ applying the solution containing the viruses to the substrate; 3‐ promoting the interaction of T4 with the substrate by vacuum drying; 4‐ gently hydrating and fixing the sample; 5‐ immunostaining.

Following phage purification using the two different protocols (see Section 4), we confirmed their presence and quality by transmission electron microscopy (TEM, Figure S1) and assessed phage concentration. We prepared the sample using phages purified by Protocol 1 due to their higher purity (Figure S1A). In this case, we labeled the phages with a solution containing T4‐specific IgG antibodies precipitated from T4‐specific serum of C57BL/6J mice (polyclonal) and secondary nanobodies conjugated to a DNA strand (Massive Photonics). We utilized a custom TIRF microscope to conduct 3D‐DNA‐PAINT imaging, employing a solution containing a complementary DNA imager tagged with the Atto 643 fluorescent dye. To achieve the 3D modality, we integrate a cylindrical lens into the imaging setup, placing it along the microscope's optical path.^[^ ^23^ ^]^ By analyzing the ellipticity and orientation changes of the fluorophore's images it was possible to determine the *z*‐coordinate of the fluorophore and thus reconstruct the corresponding 3D image.

**Figure 2:**
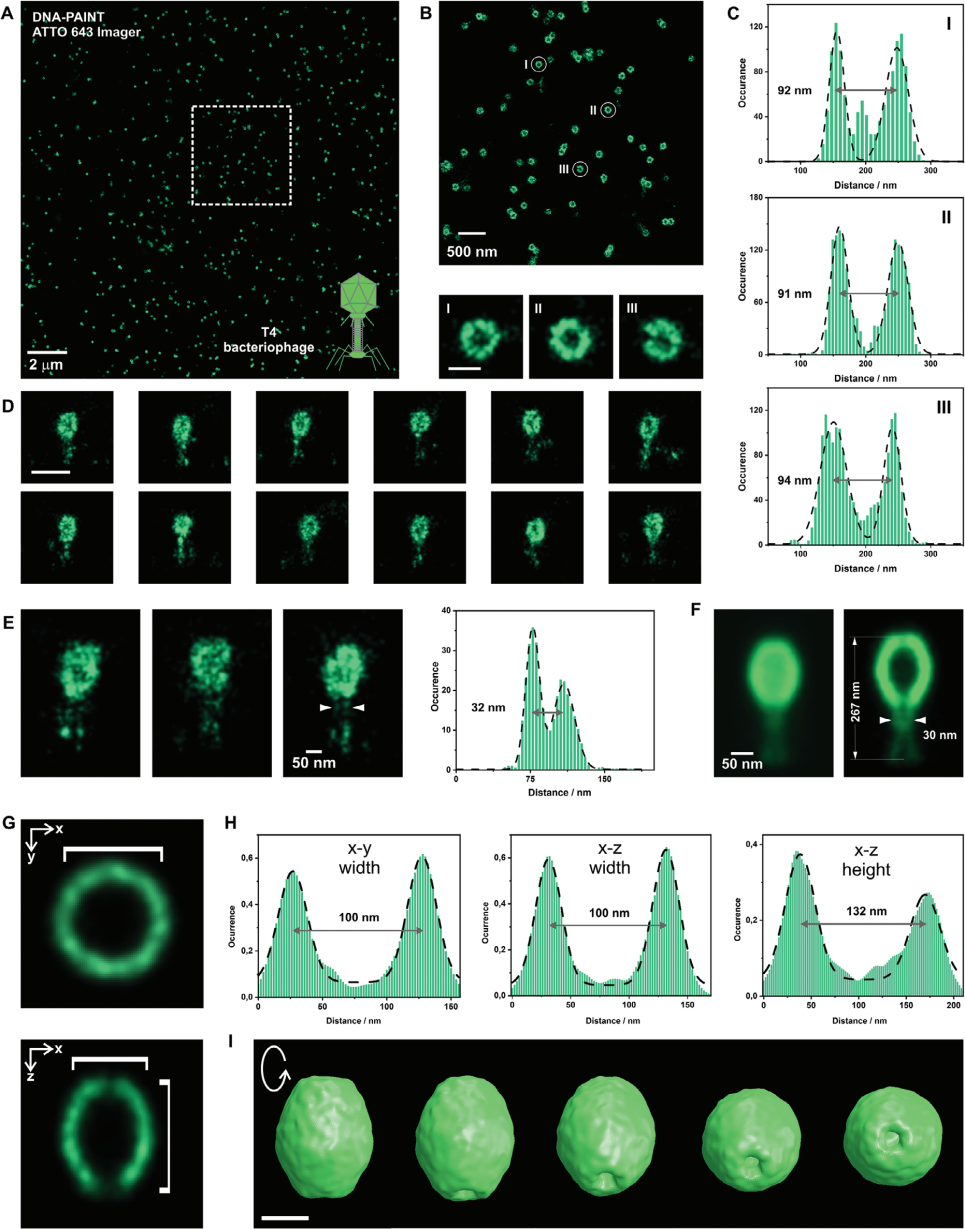
T4 bacteriophage 3D DNA‐PAINT imaging. A) Representative DNA‐PAINT super‐resolved image of the single viruses on the surface, where primary antibody recognized the whole phage. The imager was labeled with Atto 643 dye. B) *x,y –* cross sections through the center of the capsid of the region marked in (A). Several individual phages I‐III represented a hollow structure of the capsid. The scale bar is 100 nm. C) Cross‐sections through the center of the I‐III capsids. D) *xz*‐projections of the individual phages represented in (A). The scale bar is 250 nm. E) *xz*‐ projections of the individual phages where the hollow structure of the virus tail could be observed. Averaged *x,z*‐ projections of the whole phage (F) and virus capsid (G) of the dataset presented in (A). H) *x,y‐* and *x,z‐* cross sections through the center of the average capsid. I) Different views of the 3D render of the T4 average capsid presented in (G). The scale bar is 50 nm.

In **Figures** **2**, S2, and Videos S1 and S2 we show, for the first time at this level of detail for light microscopy, the 3D architecture of T4. The characteristic elongated icosahedral hollow capsid and its cylindrical tail are clearly visible. We found that with this simple protocol, hundreds of viruses were captured in sharp focus within a single field of view, with a relatively low fluorescence background, enabling measurements with a high signal‐to‐noise ratio. Strikingly, the majority of viruses (65%) were observed oriented perpendicular to the substrate, with their capsids clearly pointing upwards (Figure 2; Figures S2–S4A and Video S1 and S2). The remaining 35% of viruses were observed with the capsids touching the substrate (Figures S4A, S5), in some cases without tails. By examining the TEM images of our samples, we discovered a few cases where viruses appeared incomplete, lacked tails and fibers. This observation may explain the occurrence of some of these positions. Particularly, we estimated that ≈26% of the virus particles were aggregated as dimers, and ≈6% appeared blurred, suggesting that some particles were unstable during the acquisition. Examples of these cases are shown in Figure S6.

To determine whether the perpendicular orientation of the T4 virus was due to its interaction with the albumin substrate in our preparation, we performed a control experiment without albumin. In these samples, the viruses were vacuum‐dried directly on the glass. As expected, the majority of viruses (65%, Figure S4A,B) were in contact with the glass with their tails parallel to the substrate. Surprisingly, 35% of the viruses were oriented perpendicularly (Figure S4A,C), as in the albumin condition. However, these samples showed more contamination with small virus fragments and significant virus aggregation on the surface.

To investigate this phenomenon further and to determine whether the ability of T4 to orient perpendicularly to the substrate was specific to this phage, we used our albumin protocol with A3R, another bacteriophage from the same class (caudoviricetes) as T4. Unlike T4, A3R targets the Gram‐positive bacterium *Staphylococcus aureus*, which has a different cell envelope and receptors compared to the Gram‐negative E. coli. For this phage, we observed a similar percentage of vertically oriented viruses as for T4 on glass. (Figure S4A,E,F).

All this suggests that the ability to stand perpendicular to the coverslip is not unique to T4 and is not due to a specific interaction with albumin. However, this combination clearly favors this particular orientation.

We propose that the perpendicular orientation of the majority of T4 viruses in the albumin layer, together with their particular known geometry and asymmetry with respect to the horizontal plane (the upper part with respect to the lowest) and their versatility in handling, showcase some of the remarkable properties of this virus as a 3D‐Bio‐NanoRuler.

### 2.2 Resolution and Image Quality Estimation with the 3D‐Bio‐NanoRuler

To validate the efficacy of our T4 virus as a 3D‐Bio‐NanoRuler, we performed an analysis of the capsid size in the lateral and axial dimensions. This included determining the resolution achieved by accurately measuring the distances between the capsid walls and comparing them to the actual size. This icosahedral structure, which contains the DNA of the virus, has rounded edges and measures 86 nm in width and 120 nm in length, as determined by cryo‐EM.^[^ ^25^ ^]^ We generated 2D x,y representations from our 3D‐DNA‐PAINT data, focusing on a specific range of *z*‐positions that capture the widest span of the structure (projection depth size of 100 nm), particularly within the central region of the capsid (Figure 2). Most of the capsids appear rounded due to the projection of the majority of the icosahedral structure onto a single plane. When a smaller projection depth of 30 nm was used, a pentagon‐like structure could be distinguished in the upper or lower part of the capsid, as expected (Figure S7). Measuring the distances in the histograms of the head cross‐sections, as in Figure 2C, yielded a width of 96 ± 8 nm (mean ± SD, Figure S8), which is consistent with the expected size considering a linkage error of ≈7 nm, as previously shown for the tandem primary antibody‐secondary nanobody (for more details see Figure S9).^[^ ^26^ ^]^ Similarly, but in *x*,*z* representation, we measured the width and also the length of the capsid and obtained values of 95 ± 13 nm (mean ± SD) and 127 ± 15 nm (mean ± SD), respectively (Figure S8). These values were also consistent with the actual size of the virus head indicating that there were no substantial depth‐induced aberrations or an imperfect PSF calibration. Another notable feature of T4 is its contractile tail, which can be precisely measured. When fully extended, this appendage is 140 nm long (including the neck region) and 24 nm wide.^[^ ^27^ ^]^ In the region of the basal plate, close to the tail fibers or “legs” of the virus, its width increases to 50 nm.^[^ ^27^ ^]^ It is noteworthy that in some of the viruses observed, the tail labeling was sufficiently dense to reveal the hollow structure (Figure 2E). This appendage typically exhibited an extended length of ≈140 nm and a width of ≈30 nm in the central region, while measuring ≈49 nm in the lower basal plate segment. Furthermore, these findings also aligned with the sizes obtained by measuring a virus particle resulting from merging the localization information of 650 viruses using a particle fusion approach (Figure 2F).^[^ ^28^ ^]^ To improve the reliability of our measurements, we considered the potential tilt of some of the virus particles, a factor that could affect our results. Therefore, the analysis of the virus head was refined by aligning all particle heads to a common orientation, using the same particle averaging approach as previously used for the whole virus. This analysis revealed a prolate structure 100 nm wide and 132 nm long (Figure 2G,H, with a hole in the lower central part of the virus, consistent with the location of the tail attachment (Figure 2I; Figure S10 and Video S3). These sizes match the virus size with the expected linkage error. Furthermore, we obtained parallel results using an alternative method (Figure S10). In this approach, we align the T4 head localization point clouds based on the cryogenic electron microscopy structure provided by Fang et al.^[^ ^29^ ^]^ using the Random Sample Consensus (RANSAC) optimization algorithm.^[^ ^30^ ^]^

Finally, to evaluate the performance of our microscope, we determined the localization precision achievable in three dimensions. For this purpose, we calculated a cubic spline model of the experimentally measured PSF and generated simulated molecule images based on this model. For a range of *z* positions representing the focus depth, and for a number of photons and background level typical of our measurements (≈53 000 photons detected per DNA binding event, with 300 background photos per camera pixel) the simulated molecule images were fit using the analysis software, and the variance in the fit results yielded the expected localization precision as a function of *z* (see Figure S11). Based on these results, we expect that the typical localization precision of our data, near the focal plane, is ≈3 nm along the lateral x and y dimensions and ≈6 nm along the axial z dimension (Figure S11). This result indicates the good performance of our microscope and confirms that our 3D‐Bio‐NanoRuler measurements are within the resolution limits of our system.

### 2.3 Multi‐Target Capability of T4

A further benefit of this naturally evolved structure is the presence of the multiple protein targets that make up the phage. T4 harbours over 40 unique structural proteins that form the head, neck, tail, and fibers.^[^ ^27^ ^]^ Analyzing multiple targets within T4 potentially enables different resolution estimates or distance calibrations in multiple color channels, and also allows consistency and integrity checks by validating the correct organization of different parts of the sample structure. Here, we use “Exchange 3D DNA‐PAINT” to image first the entire virus and then a specific protein called fibritin. This protein, also known as gpwac, is a homotrimeric coiled‐coil protein that forms six 53 nm long fibers, or “whiskers,” that extend from the phage neck. This protein plays a key role in viral assembly and environmental sensing.^[^ ^31^ ^]^

In our two‐step staining procedure, we began by labeling fibritin with a specific polyclonal rabbit anti‐fibritin antibody and then labeled T4 with our previously used mouse polyclonal anti‐T4 primary antibodies. This was done to minimize the potential double labeling of the fibritin protein, as the mouse T4‐specific IgG fraction may contain anti‐fibritin antibodies. To complete the staining, we used a combination of secondary nanobodies: anti‐mouse with F1 DNA strand and anti‐rabbit with F2 DNA strand (See Section 4).

**Figure 3:**
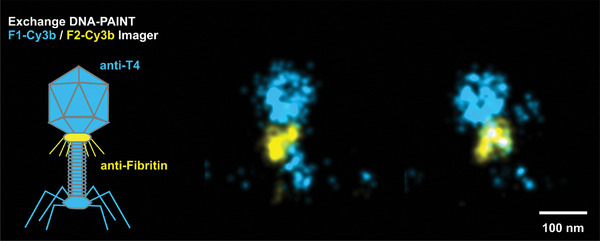
Dual color exchange DNA‐PAINT imaging of the whole T4 bacteriophage (blue) and fibritin proteins (yellow).

Our 2‐color xy plot shows the colocalization of both labeled proteins. When analyzing the 3D data, we observed the expected sub‐capsid position of fibritin and a shape that resembles the fibrous collar of this protein (see **Figure** **3**) with the expected size, indicating the reliability of the measurement.

### 2.4 Nanoruler Versatility of T4 Capsid

**Figure 4:**
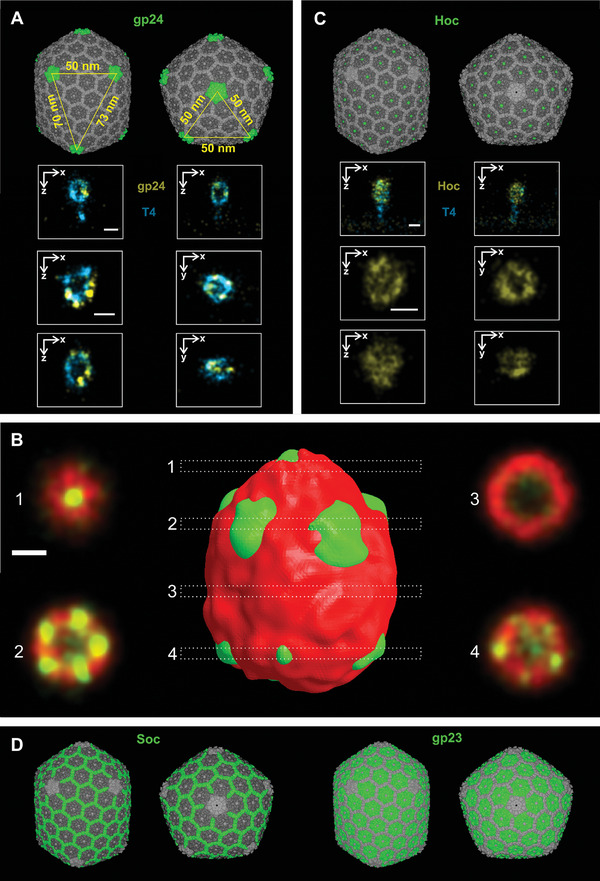
The side and top view of the T4 capsid shows in green the 4 constituent proteins that can be fluorescently labeled. A) gp24 protein: The corner position of the gp24 protein is shown, illustrating the formation of equilateral and scalene triangles (yellow). The insets show first x,z 3D‐DNA‐PAINT images of gp24 (yellow) alongside the entire T4 virus (blue), followed by x,z and x,y 3D‐DNA‐PAINT images of the capsid. B) 3D render of average capsid (red) and gp24 protein (green) and *x,y* cross sections through the entire structure. The scale bar is 50 nm. C) Hoc protein: The dotted distribution of the Hoc protein within the capsid is shown. The insets show first x,z 3D‐DNA‐PAINT images of Hoc (yellow) with the whole virus (blue), followed by x,z and x,y 3D‐DNA‐PAINT images illustrating the reticular shape derived from its labeling. D) Soc and gp23 proteins: Position of these proteins in the capsid. The scale bar size is 100 nm. The 7VS5 pdb structure was used to generate this image.^[^ ^29^ ^]^

The remarkable and structurally stable T4 capsid offers a myriad of possibilities due to its unique geometry. Consisting of a 20‐faced polyhedron with ten equilateral end cap triangles and ten scalene mid‐section triangles, it is composed of four structural proteins: gp23, gp24, Hoc, and Soc (**Figure** **4**).^[^ ^25^ ^]^ Gp23, the most abundant protein, forms hexamers that constitute the surface lattice, while gp24, the head vertex protein, forms pentamers at eleven vertices (absent only at the vertex where the tail is attached). Hoc is located at the center of each gp23 hexamer and Soc is distributed among gp23 with a hexagonal shape.

One avenue worth exploring is to use the immunolabeling of a protein such as gp24 to assess the effective labeling efficiency (ELE), in a similar way as done previously with the nuclear pore complex.^[^ ^18^ ^]^ The ELE indicates the proportion of target proteins that carry a fluorophore. This is a key factor as the accurate representation of biological samples in SMLM images is of paramount importance and is highly dependent on the density of fluorescent labeling.^[^ ^18^ ^]^ In addition, gp24 labeling may allow the measurement of smaller distances compared to the width of the capsid, facilitating higher‐resolution quantification. For example, measuring the distances between the corners of the scalene triangles (50, 70, and ≈73 nm) and the corners of the equilateral triangles (50 nm). In Figure 4A and Figure S12 we show the two types of triangles generated through 3D‐DNA‐PAINT imaging of the protein gp24. In this experiment, we also included the whole virus as a second target to better understand the gp24 positions in relation to the virus. We measured 56 ± 6 nm for the equilateral triangle sides and 72 ± 3 nm for the long scalene triangle sides, indicating a good agreement with cryo‐EM data.^[^ ^25^ ^]^ In order to obtain a more detailed view of the gp24‐labeled virus capsid and to validate the triangle measurements, we used the particle averaging method we had previously incorporated, this time focusing on the gp24 protein relative to the virus head (see Section 4). Notably, our resulting structure showed the presence of all gp24 vertices (Figure 4B), with sizes as expected: 50 nm for the equilateral triangle side and 74 nm for the scalene triangle sides.

The T4 capsid also holds the potential for measuring smaller distances, even down to the single‐digit range. For example, it's possible to label proteins such as Hoc, which maintain a separation of 14 nm between their counterparts,^[^ ^25^ ^]^ or Soc, which forms a hexagon of ≈14 nm from one side to the opposite. Another alternative is to label the six protrusions of the gp23 hexamer, which are 4.5 nm apart, or the five protrusions of the gp24 pentamer, which are 4.8 nm apart.^[^ ^25^ ^]^ In these cases, alternative labeling methods with minimal linkage error would be required. As an example, we labeled the Hoc protein using a combination of primary antibody and secondary nanobody, as described previously in this paper. Although a clearly irregular reticulated structure is visible (Figure 4B), the holes observed are larger (≈30 nm) than the expected 14 nm. This suggests under‐labeling rather than reflecting the true distances between adjacent Hoc proteins. Moreover, the linkage error of ≈7 nm of our labeling is too large to accurately visualize these tiny structures. To overcome this problem, it would be necessary to immunostain with primary nanobodies directly linked to a fluorescent probe or a DNA strand, which would significantly reduce the linkage error. Another interesting approach could be the expression of capsid proteins fused to self‐labeling tags such as HaloTag or SNAP‐tag. Notably, genetic modification of phages is well established in phage display technology, where exogenous peptides are fused to phage proteins, with T4 being one of the viruses used in this technology.^[^ ^32^ ^]^ In addition, the use of genetic code expansion for site‐specific labeling, as demonstrated with the PCNA PicoRulers,^[^ ^20^ ^]^ is proving promising. Efficient site‐specific incorporation of non‐canonical amino acids into proteins has been achieved using M13 phages.^[^ ^33^ ^]^ All these advances pave the way for extending the 3D‐Bio‐NanoRuler capabilities of bacteriophage T4.

## 3 Conclusion/Discussion

Super‐resolution fluorescence microscopy has opened unprecedented opportunities in biological research while simultaneously presenting significant challenges. One of the primary hurdles is the need to make these advanced techniques easily and reliably accessible for widespread use. To address this, the development of standard structures, or “rulers,” has become an essential strategy for the smooth adoption of these innovative methods. These reference tools, ranging from specially designed protein structures and DNA constructs to cellular architectures, offer unique benefits and face distinct limitations. However, a universally adaptable ruler—simple to produce, utilize, flexible across various experimental contexts, and effective in three dimensions—has been conspicuously missing. In this study, we present the bacteriophage T4 as a versatile and universally applicable 3D‐Bio‐NanoRuler for super‐resolution microscopy, showcasing its potential to navigate the complexities of microscopic measurement with ease and precision.

This virus, with its naturally designed protein nanostructure, is esteemed as a model organism in biological research. It has played a crucial role in enhancing our comprehension of life's fundamental principles over the years. A notable instance of its contribution is the discovery of the triplet nature of the genetic code.^[^ ^34^ ^]^ This virus, which proliferates in *E. coli*, thrives across a diverse array of environments inhabited by the bacterium, such as water bodies, soil, and even the gastrointestinal tracts of humans and animals. Recent findings have demonstrated that T4 can withstand varied temperatures and pH levels, as well as proteolytic digestive enzymes, for at least 1–2 h.^[^ ^35^ ^]^ This underscores T4's remarkable resilience across a spectrum of environmental settings, encompassing physiological, non‐physiological, and desiccated conditions.

We have devised a streamlined and user‐friendly protocol for preparing 3D‐Bio‐NanoRulers for 3D SMLM imaging. Utilizing this protocol, we have attained an unprecedented level of detail in visualizing a virus using light microscopy. Previously, 3D imaging of Alexa Fluor 647 NHS Ester‐labelled T7 phages through 4Pi single‐molecule switching nanoscopy (W‐4PiSMSN) was confined to visualizing only the virus's head. Averaging techniques later enabled researchers to discern an icosahedral structure.^[^ ^36^ ^]^ On the other hand, 2D SMLM imaging of Alexa Fluor 647 NHS‐labelled T4 facilitated a 3D phage reconstruction yet failed to reveal the hollow structures of the capsid and tail.^[^ ^37^ ^]^ In contrast, our 3D imaging technique allows for the direct observation of these distinctive hollow structures in T4 without the need for further post‐processing or analysis.

A notable finding from our protocol is that a significant number of T4 and a considerable number of A3R phages display a perpendicular orientation to the substrate, mimicking the positioning of these viruses during the infection process of their bacterial host. In this infection process, the precise factors involved in the interaction are still under investigation due to their complex and bacterial strain‐specific nature. In phages that infect *E. coli*, such as T4, there is strong evidence that electrostatic and protein‐saccharide interactions mediate binding between viral fibers and the lipopolysaccharides (LPS) on the bacterial envelope.^[^ ^38^, ^39^ ^]^ In *E. coli* K12 strain, the major receptor is the porin OmpC, which accounts for ≈50% of the protein content of the outer membrane of this bacterium. It is proposed that the ≈25 Å wide fiber ends of the phage fit into the outer cavity of the OmpC trimer and interact with its amino acids through a combination of hydrogen bonding, hydrophobic interactions, and shape‐complementary van der Waals interactions.^[^ ^38^ ^]^ On the other hand, staphylococcal phages such as A3R primarily bind to wall teichoic acids (WTAs),^[^ ^40^, ^41^ ^]^ which are anionic glycopolymers found in the cell wall of Gram‐positive bacteria. These WTAs can be further glycosylated or modified with d‐alanine residues increasing the specificity of this interaction.^[^ ^40^, ^41^ ^]^

We postulate that the perpendicular stance of T4 and A3R on the albumin substrate is favored by electrostatic attractions between the positively charged residues on the viral fibers and the negatively charged surface. BSA has been shown to possess a net negative charge at this physiological pH^[^ ^42^ ^]^ and to form monolayers with a negative zeta potential.^[^ ^43^ ^]^ The different ratios of vertical to non‐vertical orientation of these two types of phages could be due to structural differences in their fibers and the fact that A3R has a longer tail, which could destabilize the virus. Indeed, many of the standing A3R viruses were observed to be tilted (Figure S4F). Moreover, compared to the glass surface, the albumin substrate results in a much higher percentage of T4 phages in a perpendicular orientation and reduced aggregation. This difference may be due to the lower overall forces exerted by the albumin substrate. Additionally, the topography of the albumin substrate may promote shape‐complementary interactions similar to those with OmpC, which contribute to the adoption of this position.

The unique preferred perpendicular orientation of T4 in our albumin samples, previously undocumented, coupled with the virus's notable stability under various conditions, underscores its potential as a reliable and versatile 3D ruler, even within physiological environments. Additionally, phages have a documented ability to enter eukaryotic cells,^[^ ^44^, ^45^, ^46^ ^]^ a phenomenon we have observed, though only to a limited degree, even in fibroblast‐like cells (see Figure S13), which generally do not efficiently internalize T4 viruses.^[^ ^46^ ^]^ This represents a promising avenue for employing T4 as an intracellular ruler. This application warrants further investigation and optimization.

In conclusion, the T4's exceptional 3D orientation, combined with the capability to estimate nanometer‐scale resolution in all dimensions and to detect multiple targets, consolidate the T4's position as a state‐of‐the‐art 3D Bio‐NanoRuler with remarkable versatility.

## 4 Experimental Section

### Bacteriophages

T4 phage was obtained from the American Type Culture Collection (ATCC, Rockville, MD, USA) and propagated on *Escherichia coli B* host from the Polish Collection of Microorganisms (PCM). A3R phage was obtained from the Therapeutic Phage Collection of the Hirszfeld Institute of Immunology and Experimental Therapy (HIIET) Polish Academy of Sciences (PAS) and propagated on *Staphylococcus aureus* strain (PCM) isolated from patients of the Phage Therapy Unit at the HIIET PAS.

All phage lysates were prepared in enriched nutrient broth inoculated with an appropriate overnight bacterial host suspension. After culturing *Staphylococcus aureus* for 0.5–1 h at 37 °C, A3R was added at a final titer of 10^6^ pfu mL^−1^, and the culture was kept at room temperature for 30 min to allow for phage adsorption. The *Staphylococcus aureus* culture supplemented with A3R was then incubated at 37 °C with vigorous shaking for 7 h and transferred to 4 °C for 2 days to clarify. In the case of T4 phage lysate preparation, *Escherichia coli* B was grown at 37 °C to OD_600_ = 0.6 and then T4 phage was added at a final titer of 10^6^ pfu mL^−1^. The T4‐supplemented *Escherichia coli* B culture was then incubated at 37 °C with vigorous shaking for 8–10 h and transferred to 4 °C for 2 days for clarification.

All phage lysates were centrifuged at 7000 g, 4 °C for 8 min, and the supernatants were filtered through 0.22 µm Millipore PES membrane filters (Merck Millipore). Filtered supernatants were processed with a Sartorius Hollow Fiber (HF) 115 cm^2^ cutoff 750 kDA cartridge at a pressure lower than 0.68 Ba at room temperature. Buffer exchange from growth medium to PBS was performed by processing material with five diavolumes of the buffer. Next, phage preparations were concentrated using the same HF system at the same pressure force, reducing the buffer volume from 300 to 40 mL.

In addition, the T4 phage preparation was purified by LPS‐affinity chromatography using EndoTrap HD (Lionex GmbH, Germany). At the end of the process, all phage preparations were dialyzed against PBS using 1000 kDa‐pore membranes and filtered using 0.22 µm PES filters (Merck Millipore, Billerica, MA, USA).

### Bacteriophages Quantification

The purified phage preparation was assessed for phage concentration by determining the phage titer through serial dilution with PBS. 50 µL of each dilution was spotted on a culture plate pre‐covered with susceptible bacteria, with three spots for each dilution. The plate was incubated at 37 °C for 8–10 h until visible plaques appeared. The plaques were counted, the mean values of three spots were calculated, and the phage concentration per milliliter was determined considering the dilution and spot volume.

### TEM Imaging

A droplet of bacteriophage solution (at a concentration of 10^12^ PFU mL^−1^) was carefully absorbed onto carbon‐coated copper grids (300 mesh, Ted Pella, Inc.), followed by a gentle wash with Milli‐Q water. The grids were then stained with a 1% (w/v) uranyl acetate solution before imaging with a CM30 LaB6 transmission electron microscope (Philips).

### Bacteriophage‐Specific Serum Production

C57BL/6J normal male mice (Mossakowski Medical Research Centre, Polish Academy of Sciences, Warsaw) were challenged with purified T4 or A3R phage to obtain phage‐specific serum. Three doses of highly purified phage preparation were administered intraperitoneally (IP) at 10^10^ pfu per mouse on days 0, 20, and 50. Blood samples were collected from the tail vein on day 100 and serum was separated as follows.

Serum was collected in tubes (BD SST II Advance), allowed to clot for 1 h at room temperature (RT), and then separated from the clot by centrifugation (10 min, 2000 g). The separated serum was stored at −20 °C until further use. The presence of phage‐specific IgG antibodies in the serum was confirmed by ELISA.

### Proteins and its Serum‐Specific Production

The phage protein gp24 and Hoc produced as described by Miernikiewicz et al.^[^ ^47^, ^48^ ^]^ Briefly, the gene encoding the mature form of the proteins was cloned into pDEST15 using Gateway technology, allowing the expression of recombinant proteins with a glutathione S‐transferase (GST) affinity tag. Both proteins were expressed in the *Escherichia coli* B834 (DE3) expression system. For expression of Hoc chaperones groES+groEL (from pGRO7 vector, TaKaRa Bio Inc., Saint‐Germain‐en‐Laye, France) were used. Expression of the recombinant phage proteins was induced with 0.2 mm isopropyl‐β‐d‐1‐thiogalactopyranoside (IPTG) and carried out overnight at 25 °C. Harvested bacteria were lysed with lysozyme in phosphate buffer containing phenylmethylsulfonyl fluoride (PMSF) (50 mm Na_2_HPO_4_, 300 mm NaCl, 1 mm PMSF; pH 7.5) and freeze‐thawed. The soluble fraction was incubated with glutathione sorbent slurry (Glutathione Sepharose 4B; GE Healthcare Life Sciences), washed with phosphate buffer, and phage proteins were released by proteolysis with AcTev protease (5 U mL^−1^) (Invitrogen, Life Technologies Corporation) at 10 °C. Lipopolysaccharide (LPS) was then removed using EndoTrap HD (Lionex GmbH, Germany), and gel filtration fast protein liquid chromatography (FPLC) was performed on a Superdex 75 10/300 GL column (GE Healthcare Life Sciences). The sample was dialyzed against PBS and filtered through 0.22‐µm polyvinylidene difluoride (PVDF) filters (Millipore). Protein concentration was determined by the Lowry chromogenic method (Fermentas International Inc.).

To induce anti‐gp24 and anti‐Hoc antibodies in mice (polyclonal), male C57BL/6J mice (Mossakowski Medical Research Centre, Polish Academy of Sciences, Warsaw) were separately challenged subcutaneously with three doses of the previously purified gp24 or Hoc protein (200 µg per mouse) on days 0, 20, and 40. Blood was collected from the orbital vein up to 7 days after the last dose.

Serum was collected in tubes (BD SST II Advance), allowed to clot for 1 h at RT, separated from the clot by centrifugation (10 min, 2000 g), and stored at −20 °C until further use. The presence of protein‐specific IgG antibodies in the serum was confirmed by ELISA.

### Ammonium Sulfate Antibody Precipitation

T4‐specific and gp24‐specific serum samples were incubated in a 50% ammonium sulfate saturated solution for 30 min on ice. The suspension was then centrifuged at 10 000 g for 12 min at 2 °C. The supernatant was removed, and the pellet was resuspended in PBS and stored at −20 °C until further use.

### Bacteriophages Sample Preparation

Cleaned 18 mm coverslips (Paul Marienfeld, 0117580) were used directly (in the “glass” substrate) or coated with 5% BSA solution in PBS (Blocking solution) for 2 h at RT or overnight at 4 °C (in the “albumin” substrate). The albumin‐coated glasses were washed three times with PBS before use. Subsequently, 10 µL of T4 or A3R in PBS (8 × 10^11^ PFU mL^−1^) was applied to the wet slides. The coverslips were then vacuum dried for 10 min in a centrifuge concentrator (Concentrator plus, Eppendorf) without rotation. The samples were then simultaneously hydrated and fixed with a solution of 4% PFA in PBS for 20 min, or alternatively gently hydrated with PBS for 5 min followed by the addition of 16% PFA solution in PBS to a final concentration of 4% PFA and incubated for 20 min. An optional quenching step was performed with 0.1 m glycine in PBS for 10 min. Finally, the coverslips were washed three times with PBS and blocked with Blocking solution for 1 h at RT.

### Bacteriophages Immunolabeling

After blocking, T4 and A3R samples were incubated with a 1:30 dilution of mouse T4‐ or A3R‐specific IgG antibodies in a blocking solution for 1 h to achieve single target virus labeling. For dual target labeling in T4, staining was performed in two steps: T4 samples were first incubated with a 1:50 solution of rabbit anti‐wac (fibritin) antibody (2.95 mg mL^−1^; CUSABIO, No. CSB‐PA319157ZA01EDZ), or anti‐gp24 or anti‐hoc in Blocking solution for 1 h, followed by incubation with a 1:30 solution of mouse T4 specific IgG antibody in blocking solution for 1 h. The samples were washed three times for 10 min each with PBS and then incubated for 45 min with a mixture of 1:50 DNA strand conjugated single domain secondary antibodies (FluoTag‐XM‐QC anti‐mouse IgG kappa light chain + FAST docking site F1 and FluoTag‐XM‐QC anti‐rabbit IgG + FAST docking site F2; Massive Photonics) in a solution of 5% BSA in PBS. The samples were then washed three times for 10 min each with PBS before imaging. An optional post‐fixation step with 4% PFA (Sigma–Aldrich, F8775) in PBS for 10 min was performed in cases of long‐term sample storage.

### COS‐7 Cell Culture and T4 Uptake Experiment

COS‐7 African green monkey fibroblast‐like cells were cultured in T25 flasks at 37 °C in 5% CO_2_. The growth medium used was Dulbecco's modified Eagle's medium (DMEM) supplemented with 10% fetal bovine serum (Sigma–Aldrich, F7524), 100 U mL^−1^ penicillin and 0.1 mg mL^−1^ streptomycin (Sigma P4333). One day before use, cells were seeded onto 18 mm coverslips (Paul Marienfeld, 0117580) at a density of 10^5^ cells coverslip and incubated overnight. The next day, the cells were exposed to 20 µL of T4 in PBS (8 × 10^11^ PFU mL^−1^) and fixed with 4% PFA after an incubation of 30 min.

### COS‐7 Cells Immunolabelling

After fixation, cells were washed twice with PBS and quenched with 0.1 m glycine for 10 min. Afterward, cells were washed three times with PBS before blocking and permeabilizing with 5% BSA and 0.1% Triton X‐100 for 30 min. The cells were then washed again with PBS and incubated with primary antibodies. The primary antibody solution consisted of a 1:100 dilution of anti‐TOMM20 antibody (Abcam, ab186735) and a 1:30 dilution of anti‐T4 antibody, both in 5% BSA in PBS. After primary antibody incubation, samples were washed three times for 10 min each with 0.1% Tween‐20 in PBS. The cells were then incubated for 45 min with the same mixture of DNA strand conjugated single domain secondary antibodies used in *Bacteriophages immunolabeling*. Finally, the samples were rinsed in PBS three times for 10 min before imaging.

### DNA‐PAINT Imaging

3D DNA‐PAINT imaging was performed on the Olympus IX‐71 based on an inverted microscope stand and equipped with a UPLANSAPO 100X NA 1.4 oil‐immersion objective lens. Laser illumination sources used for SMLM imaging included red and green lasers for imaging (642 nm CW, 1.5 W, MPB Communications Inc.; 532 nm CW, 1.2 W, Light Cube LC‐LS‐532‐1.2 W). Excitation light was controlled and modulated via an acousto–optic tunable filter (Crystal Technologies, AOTF‐PCAOM). Variable angle TIRF or near‐TIRF illumination was achieved using a custom light path entering through the rear port of the microscope. Excitation light was separated from fluorescence using a dichroic beam‐splitter in the filter cube turret (650DCXR or Z532RDC, Chroma Technology Corp.) and an emission filter in the detection path (ET700/75 or HQ582/75, Chroma). Fluorescence light was collected by the objective lens, passed through an optical relay, and focused to form an image on a back‐illuminated EMCCD camera (Andor Ixon+, DU860). For 3D imaging, a cylindrical lens (*f* = 500 mm) was placed in the optical path before the image plane. The microscope was equipped with a motorized sample stage (Märzhäuser Wetzlar), and the objective lens was mounted on a piezoelectric objective positioner (Piezo Jena). During image acquisition, the objective *Z* position was continuously adjusted to maintain a constant focus position. This focus‐lock system was based on an infrared laser beam introduced into the microscope via the right‐side port below the filter turret and combined into the optical path using a short‐pass dichroic mirror (900DCSP, Chroma). The focus lock laser (980 nm, Thorlabs) was aligned to focus at the back focal plane of the objective lens and reflect from the glass–water interface of the sample. The position of the reflected beam was detected using a quadrant photodiode (Silicon Sensor Intl., QP50) which was monitored via a DAQ card (National Instruments). All microscope control and data acquisition were performed using custom software written in Labview (National Instruments). The sample was illuminated with 642 or 532 nm excitation light. The emitted light was filtered spectrally (see above) and detected at the EMCCD camera, running at a frame rate of 50 Hz. Typically, ≈100 000 image frames were acquired in a single measurement. Optical stabilization of the *z*‐focus (focus‐lock) was engaged before starting each recording, in order to minimize sample drift during the measurement. Prior to the measurements, images of a fluorescent bead located on the sample were recorded as the bead was scanned in the *Z*‐dimension, in order to create a calibration scan which was used in post‐processing analysis of the image data.

For the single‐target scenario, imaging was performed for 2 h using the imaging buffer from the MASSIVE‐SDAB‐FAST 2‐PLEX kit (Massive Photonics) containing 250 pm Atto 643‐labelled Imager F1 supplemented with 80 nm gold nanoparticles (BBI Solutions) at a 1:10 dilution for drift correction. Exchange‐DNA‐PAINT strategy was utilized for the two‐target scenario. First, the imaging buffer containing 250 ps Cy3b‐labelled Imager F1 together with the gold nanoparticles was added and imaged for 2 h. The sample was then carefully washed two times with 500 mm NaCl PBS (pH 8.0) and the imaging buffer containing 250 pm Cy3b‐labelled Imager F2 was added and imaged for a further 2 h.

### Data and Statistical Analysis

2D and 3D SMLM data were analyzed using custom software packages written by Dr. Mark Bates (unpublished). In general, SMLM image analysis and reconstruction follow a standard approach based on peak finding and localization. Correction of sample drift in post‐processing was done based on image correlation of the SMLM data with itself over multiple time windows, using the COMET method (Reinkensmeier et al., manuscript in preparation). SMLM images were rendered as summed Gaussian spots (2D imaging) or as localization histograms (3D imaging) with a bin size typically chosen to be 6 nm. 3D data video (see Supplementary Information) was performed utilizing ViSP.^[^ ^49^ ^]^

Viruses were counted by manually selecting xy images showing capsids in contact with the surface for non‐standing viruses and those with capsids ≈200 nm above the surface for standing viruses. The virus particle images were analyzed using a custom algorithm tailored to identify the toroidal feature characteristic of the hollow virus capsid and accurately select those corresponding to the virus size. The number of particles found in a dataset corresponding to a virus in a given state ranged from 426 to 1074 with a mean of 677. The result was presented as a percentage of the total number of viruses within the selected group (Figure S2). To analyze virus capsid size, *XY* and *XZ* intensity profiles of the virus capsids were used and the resulting curves were fitted with Gaussian peaks. The distances between the peaks were measured to reflect the sizes of the viruses. Any fits with artifacts were excluded and outliers were filtered out using a modified *Z*‐score approach. The size of the sample after filtering was 239 elements for head width in *XY* projection, 72 elements for head width in *XZ* projection, and 56 elements for head length in *XZ* projection. The resulting data was presented as sample mean value and standard deviation (Figure S5).

The side distances of the gp24 triangles were measured in 3D from 30 scalene and 30 equilateral triangles as follows: The localization cloud of each gp24 protein was analyzed to find the center of mass. This center of mass represents the average position of the protein, derived from all the localizations in the cloud. Calculating the center of mass reduces the uncertainty in protein position and provides a single, accurate 3D coordinate for each gp24 protein. Once the center of mass was determined for each gp24 protein, the 3D distances between pairs of proteins were calculated using their center coordinates. These distances provide accurate inter‐protein measurements and allow structural insights into the gp24 protein arrangement within the T4 virus.

To accurately align all the virus particles, two complementary approaches were used. The first method involved particle averaging, a technique also known as Particle fusion.^[^ ^50^ ^]^ The second approach utilized a model of an expanded virus head as the reference for alignment. This strategy combined global registration using the RANSAC algorithm^[^ ^30^ ^]^ with local registration through the Iterative Closest Point (ICP) algorithm,^[^ ^51^ ^]^ both of which were implemented in the Open3D Python package.^[^ ^52^ ^]^

The virus head model was derived from an expanded T4 virus structure (https://doi.org/10.2210/pdb7vs5/pdb),^[^ ^29^ ^]^ initially containing a large number of atoms. To optimize the analysis, nitrogen atoms were selectively isolated and the resulting point cloud was subsequently downsampled.

To mitigate potential biases, a 17 nm thick slice of the point cloud was sliced through the center in both *xy* and *xz* planes. From these images, 17 nm thick stripes were further selected to generate an intensity profile. Using a least‐squares objective function and the L‐BFGS‐B optimization procedure, two Gaussian peaks were fitted to the profile. The centers of these peaks were then recalculated into distance measurements. The total number of particles analyzed with these methods was 126.

In order to analyze the gp24 localization set, the same particle averaging technique was employed. Recognizing the limitations imposed by low statistical representation, a guided alignment approach was adopted. The existing dataset of 56 virus particles was augmented by introducing six additional model particles (representing a 10% increase). These new particles comprised both gp24 points and virus head points. The analysis was conducted using this expanded set and subsequently, the model particles were removed from the final dataset.

### Ethics Statements

All animal experiments were performed according to EU Directive 2010/63/EU for animal experimentations and were approved by the 1st Local Committee for Experiments with the Use of Laboratory Animals, Wroclaw, Poland (No. 64/2009 and 76/2011). The authors followed the ARRIVE (Animal Research: Reporting of in vivo Experiments) guidelines.^[^ ^53^ ^]^

## Conflict of Interest

The authors declare no conflict of interest.

## Author Contributions

J.I.G., O.N., M.B., and J.E. conceived the project. J.I.G. prepared the virus and cell samples. M.B. designed and constructed the 3D STORM microscope and created the microscope control and data acquisition software. M.B. created the data analysis software. Imaging and data analysis were performed by J.I.G., O.N., and M.B. J.I.G. wrote the manuscript and O.N. prepared the figures. ZK, PM, and KD contributed to virus solutions and antibody production. Electron microscopy images were obtained by T.C. and A.C. I.G. and L.R. assisted with data visualization and analysis. The project was led by J.I.G., O.N., M.B., and J.E. All authors have revised and approved the manuscript.

## Supporting information



Supporting Information

Supplemental Movie 1

Supplemental Movie 2

Supplemental Movie 3

## Data Availability

The data that support the findings of this study are available from the corresponding author upon reasonable request.
