# Supplementary material for: Super‐Resolution Goes Viral: T4 Virus Particles as Versatile 3D‐Bio‐NanoRulers
Source: Adv Mater. 2025 Jan 16;37(12):2403365. doi: 10.1002/adma.202403365 (PMC11937993; doi:10.1002/adma.202403365)
Supplement: Supplementary file 1 — Supporting Information [file ADMA-37-2403365-s001.docx]

Supporting Information

Super-resolution goes viral: T4 virus particles as versatile 3D-Bio-NanoRulers

José Ignacio Gallea,* Oleksii Nevskyi,* Zuzanna Kaźmierczak, Ivan Gligonov, Tao Chen, Paulina Miernikiewicz, Anna Chizhik, Lenny Reinkensmeier, Krystyna Dabrowska, Mark Bates,* and Jörg Enderlein*


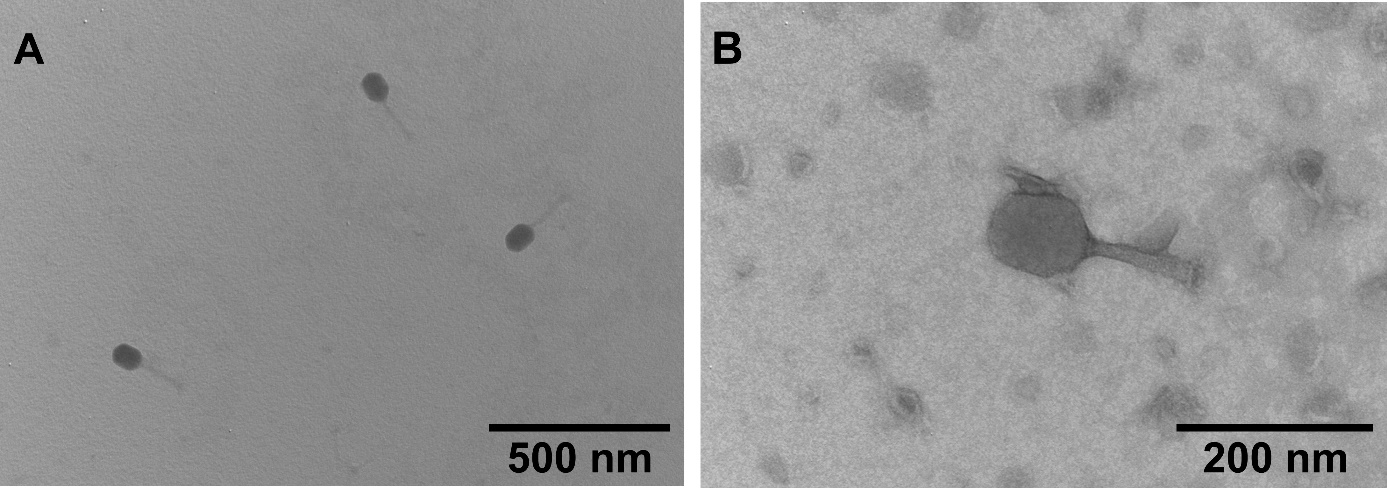


Figure S1: TEM images of T4 viruses purified by protocol 1 (A) and protocol 2 (B).


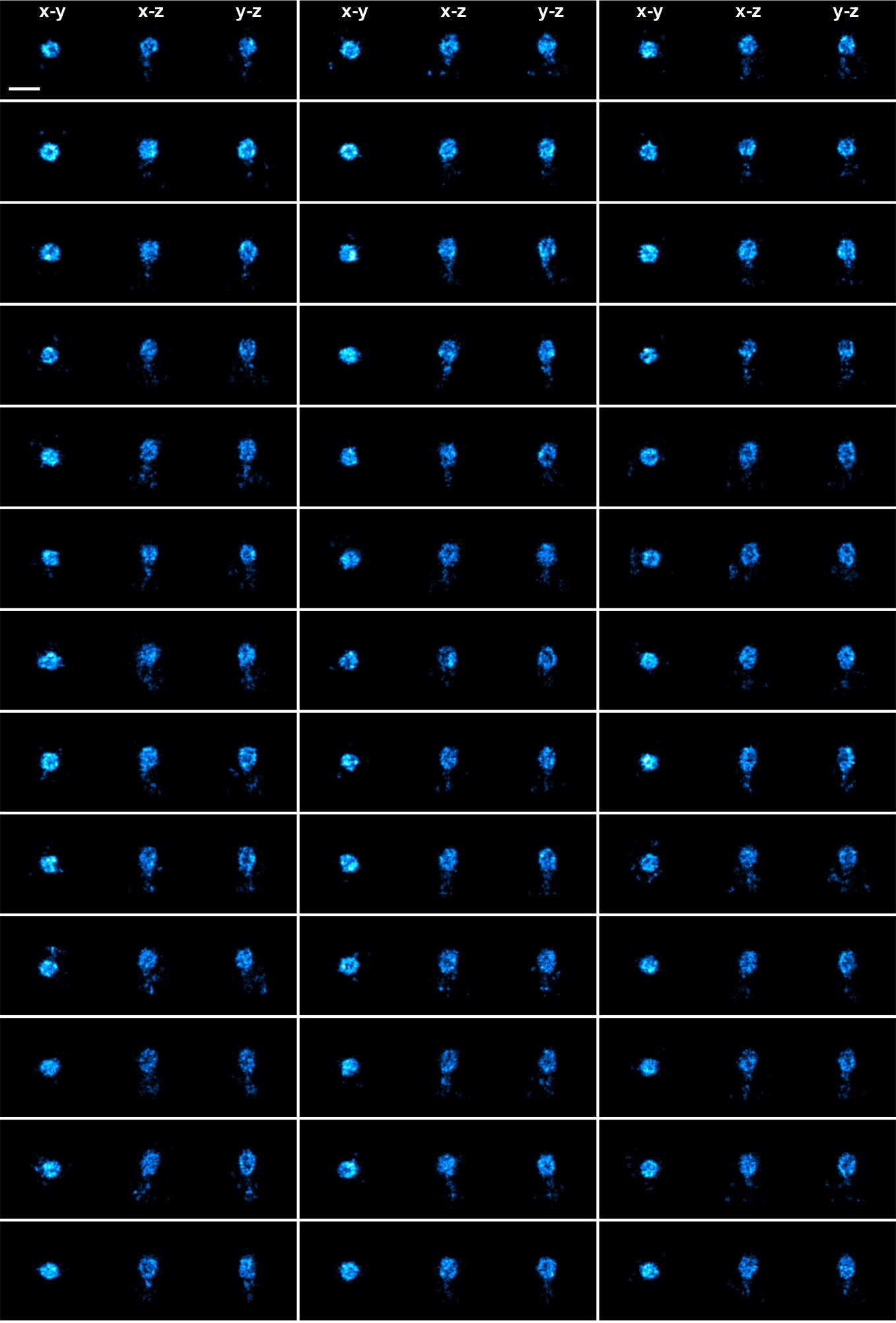


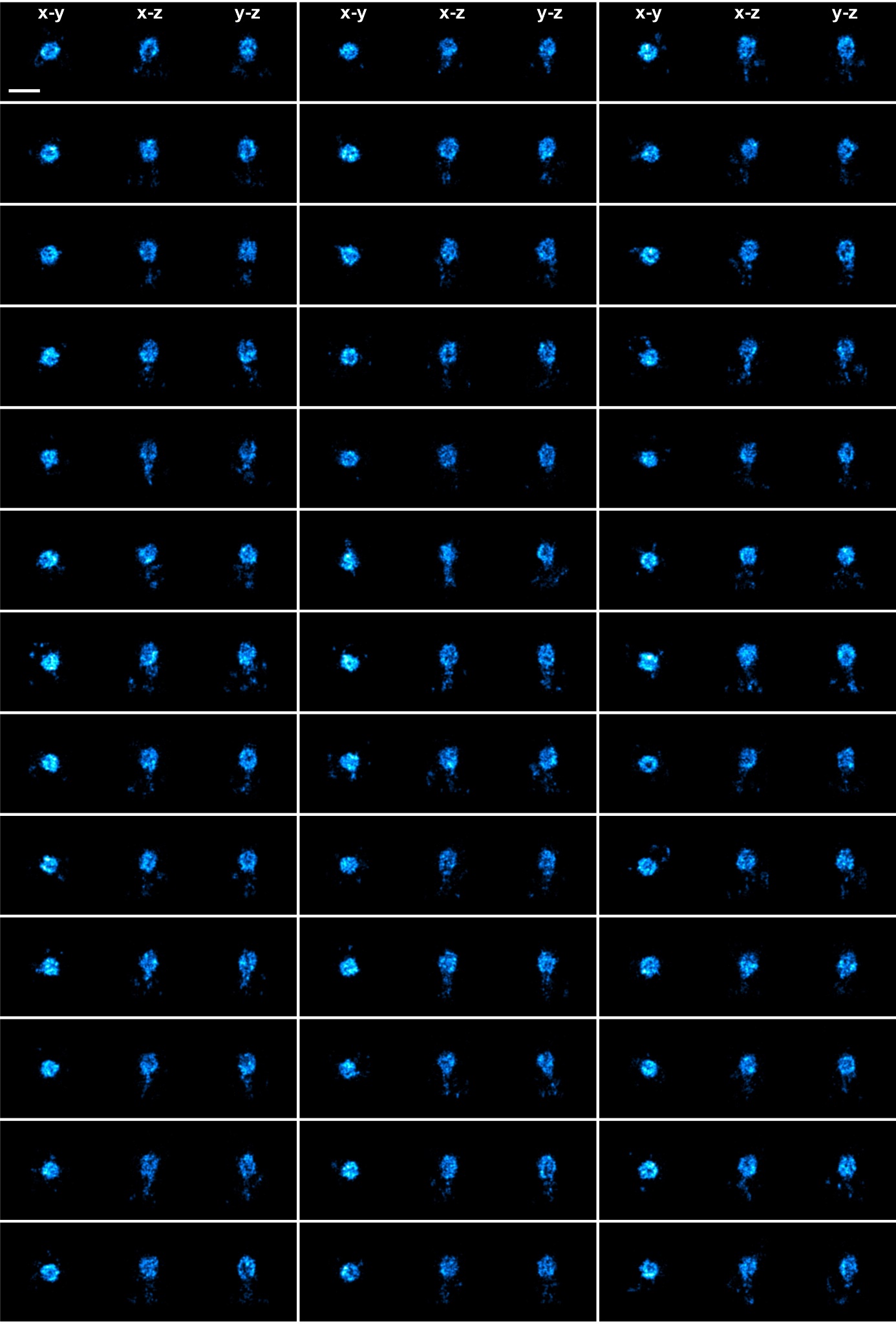


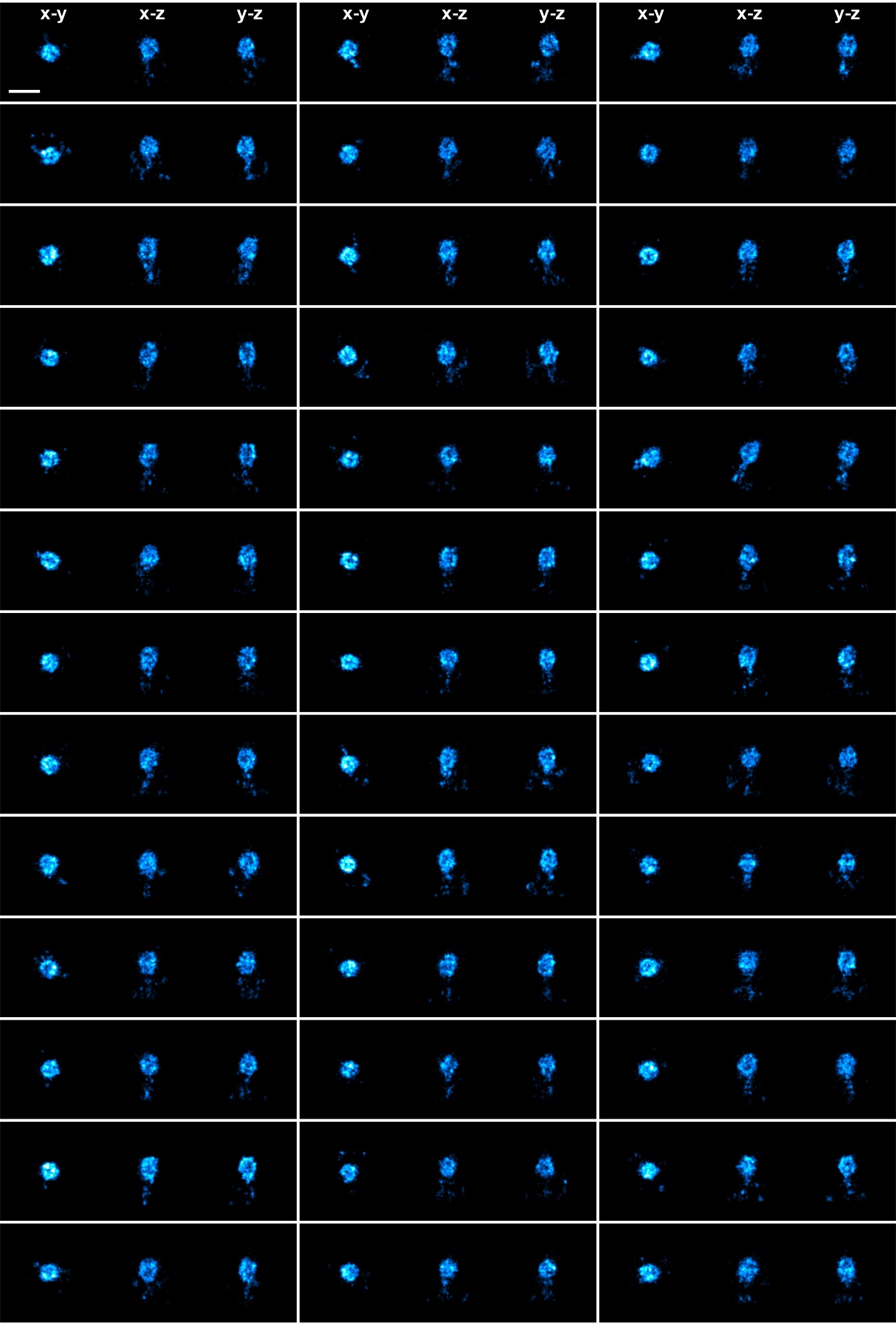


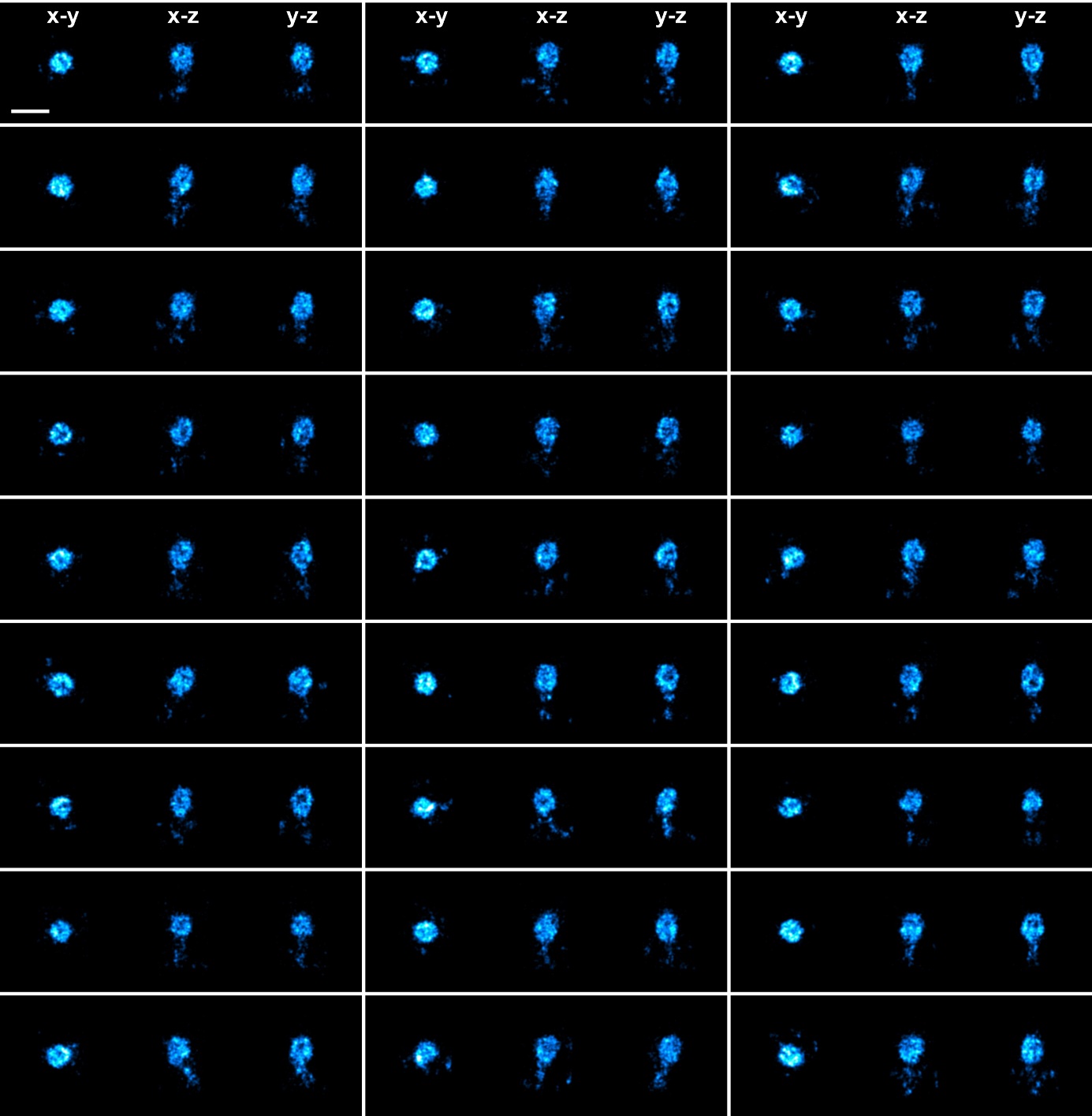
Figure S2: Examples of 3D DNA-PAINT images of 144 T4 viruses on an albumin surface labelled with T4-specific IgG antibodies. The xy, xz and yz projections are shown for each virus. Scale bars are 200 nm.


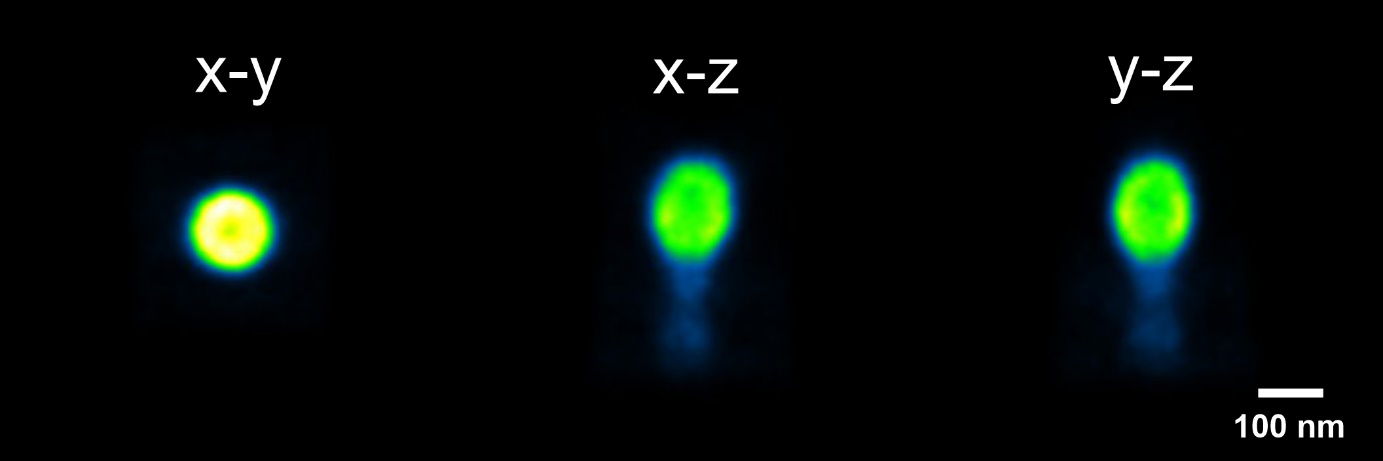


**Figure S3: Average projections of the 144 T4 images in Figure S2.**


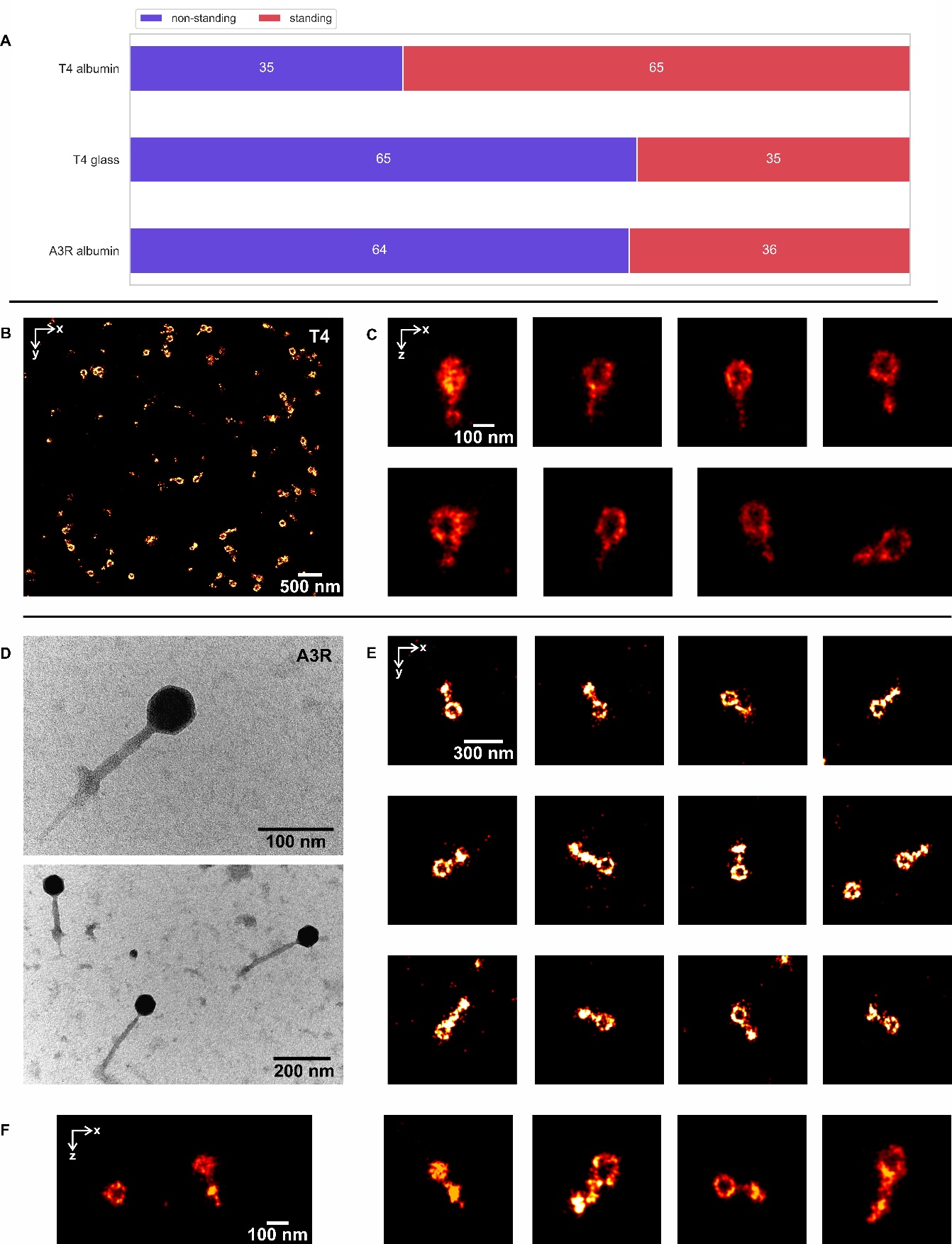


Figure S4: A) Percentages of viruses standing perpendicular to the surface versus not standing obtained from five independent experiments. B) *xy* 3D-DNA-PAINT image of T4 virus on a glass surface. C) *xz* 3D-DNA-PAINT images of T4 virus on a glass surface. D) TEM images of A3R phages. E) *xy* 3D-DNA-PAINT images of A3R virus on an albumin surface. F) *xz* 3D-DNA-PAINT images of A3R virus on an albumin surface.


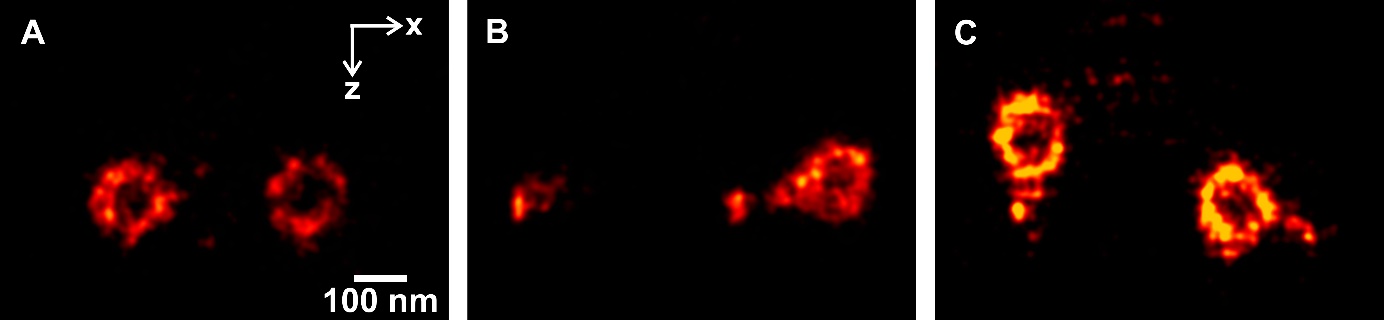


**Figure S5: *xz* 3D-DNA-PAINT images showing examples of broken (A, B) / underlabeled
(C) T4 viruses.**


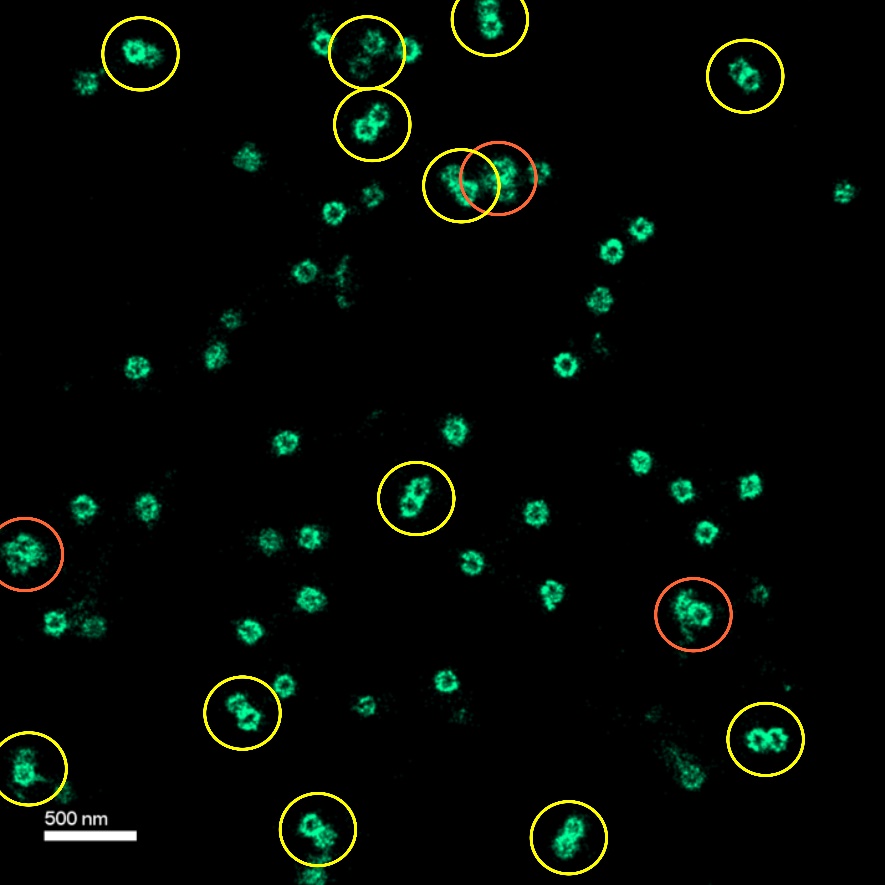


Figure S6: *xy* 3D-DNA-PAINT images of T4 viruses, highlighting distinguishable virus dimer aggregates (yellow circles) and unstable blurred viruses (red circles).


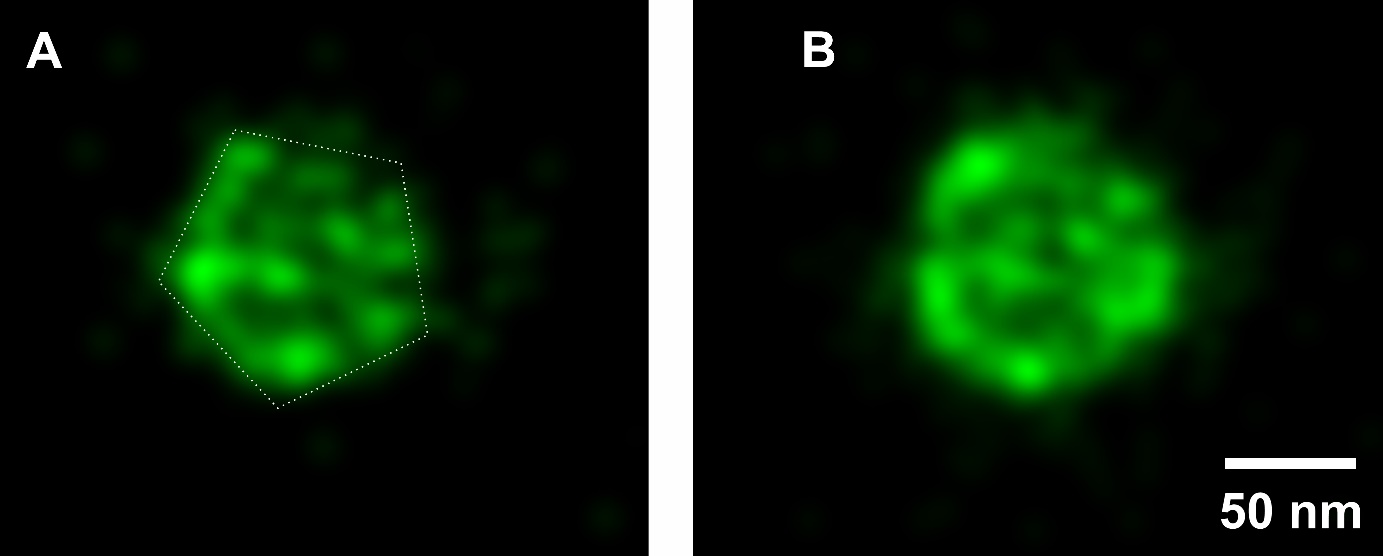


Figure S7: *xy* cross-section of the upper part of the phage head taken with at a projection depth of 30 nm (A) and 100 nm (B).

**
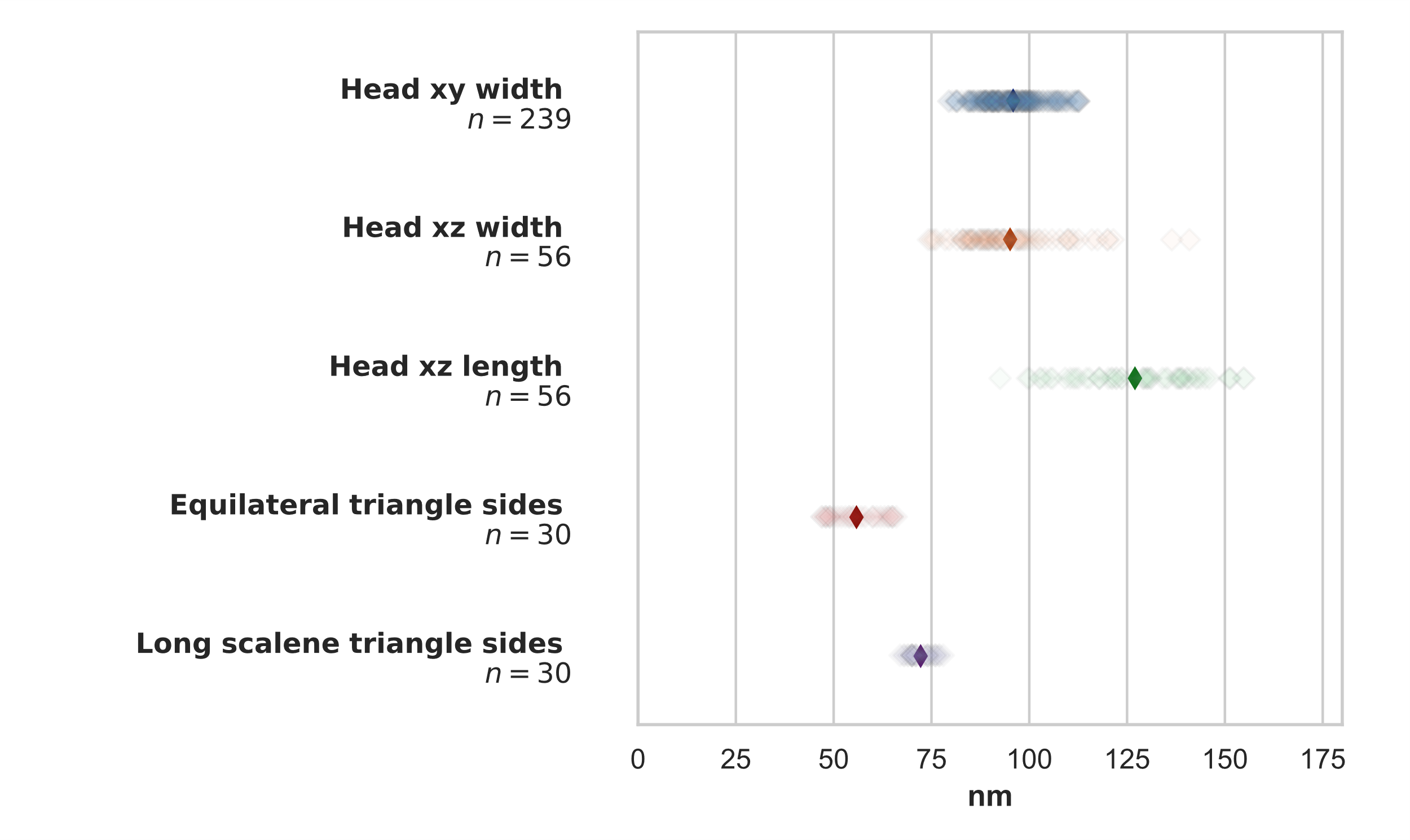
**

**Figure S8: Measurements of T4 capsids obtained from 3D-DNA-PAINT images in five independent experiments. Mean values are shown as opaque diamonds. Measurements yielded values of 96±8 nm for capsid width in *xy*, 95±13 nm for capsid width in *xz* and 127±15 nm for capsid length in *xz* (mean ± SD). Additionally, the gp24 protein triangle sides were measured to be 56 ± 6 nm (equilateral) and 72 ± 3 nm (scalene).**

**
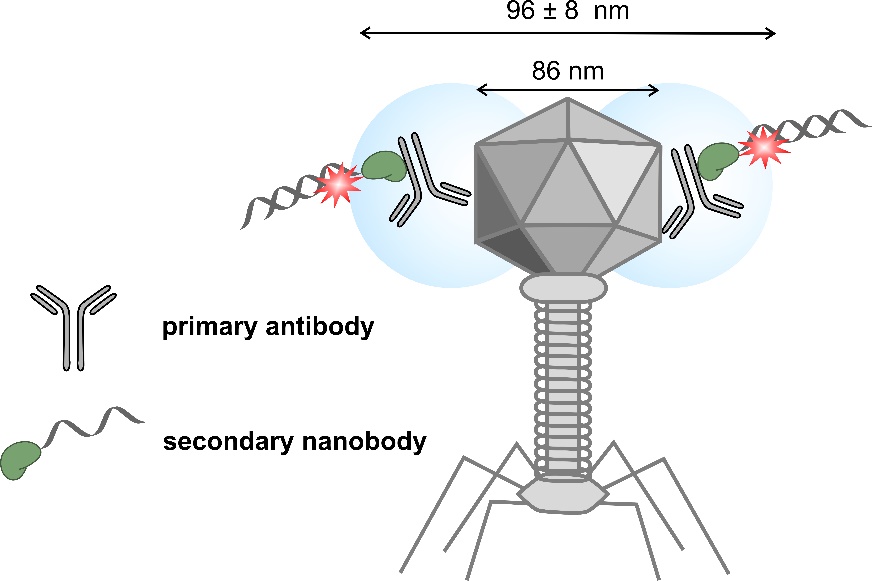
**

**Figure S9: Illustration of linkage error in immunolabeling using a primary antibody and secondary nanobody.** **The image depicts the spatial displacement between the target and the fluorescent marker due to the size and the orientation of the primary antibody and the secondary nanobody. The total linkage error is ~ 5 nm when considering that both sides contribute to this distance.^[24]^**


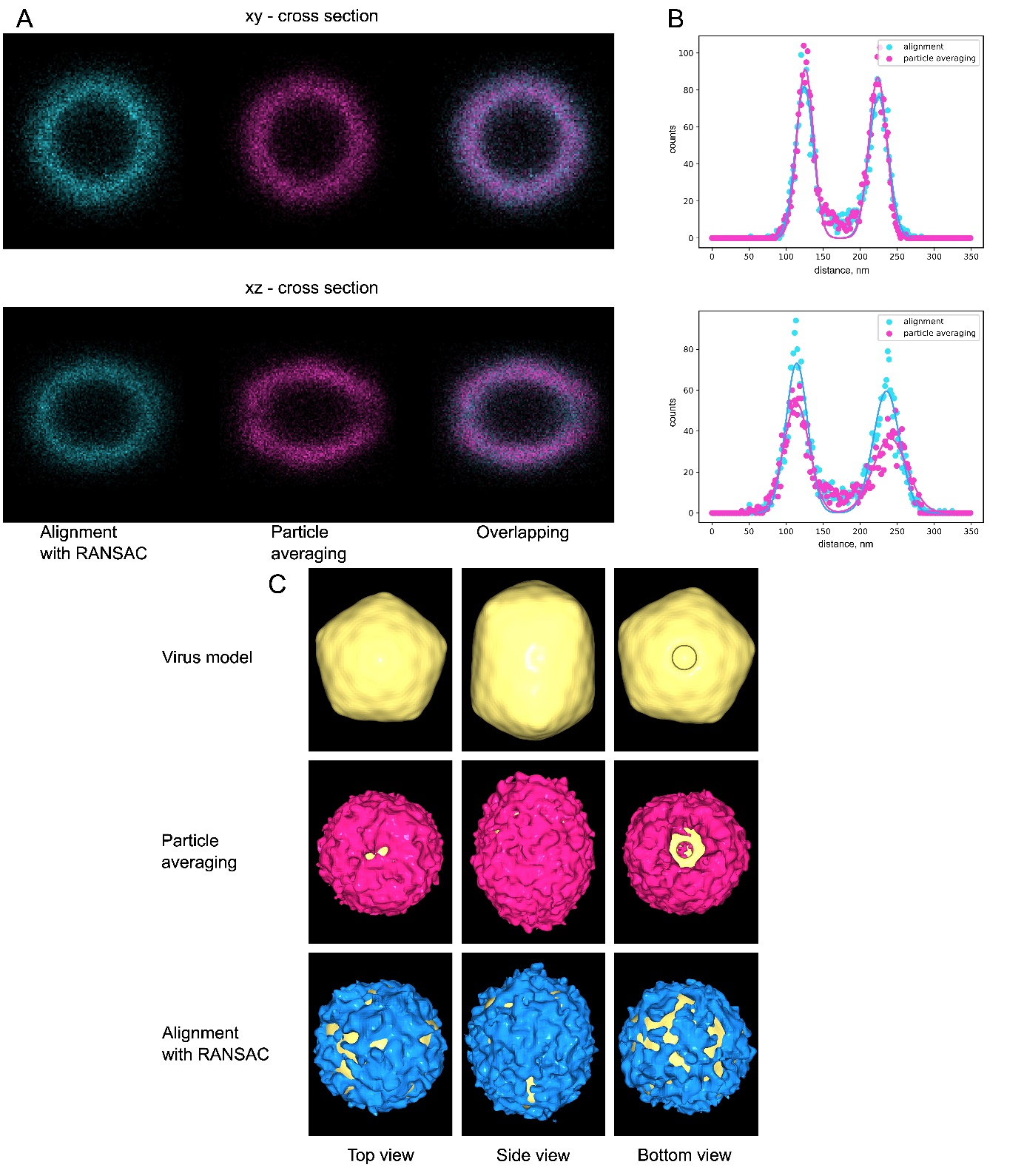


**Figure S10: Comparison of alignment methods for T4 head. (A) Cross sections of aligned point clouds in *xy* and *xz* planes and their overlap. (B) Cross section profiles. The curves were fitted with two gaussians yielding the distances: in *xy* – 100 and 98 nm, in *xz* – 122 and 126 nm. (C) The rendering of point cloud densities as isosurfaces and comparison with the model of T4 virus head.**


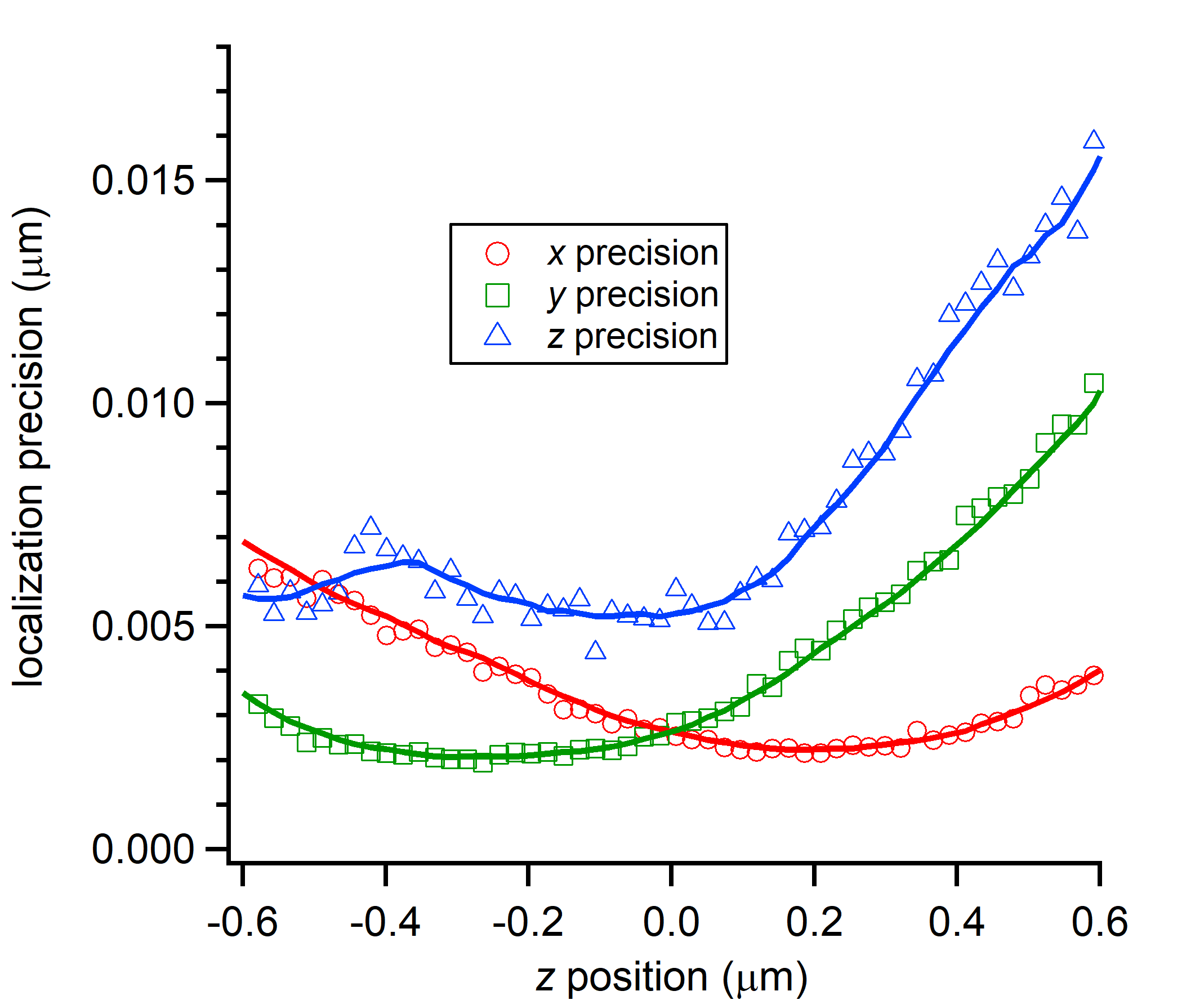


**Figure S11: Localization precision analysis. Based on the experimentally determined PSF, we calculated the expected localization precision of the measurement according to the number of photons detected per DNA-PAINT binding event, and the fluorescence background level of the typical measurement. Based on a cubic spline model of the measured PSF, single molecule images were simulated for different *z* positions in the sample. For each *z* position, the measurement error was determined by comparing the fit results with the simulation ground truth. Simulation data points are shown as markers, and solid lines indicate the smoothed data. The results show that, near the focal plane, the microscope achieves a localization precision of approximately 3 nm in the lateral dimensions (*x,y*) and 6 nm along the axial direction (*z*).**


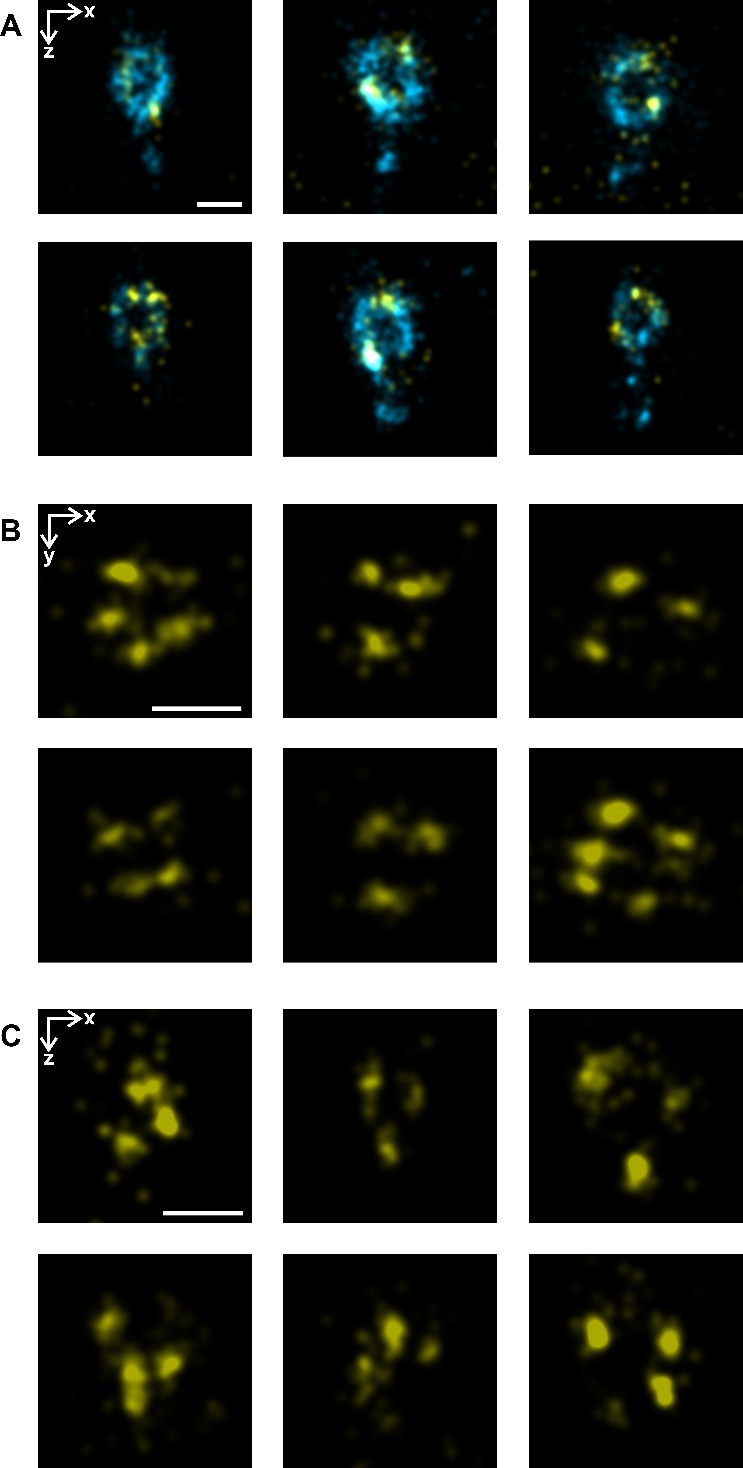


Figure S12: A) *xz* 3D-DNA-PAINT images showing gp24 protein (yellow) together with the whole T4 virus (blue). B) *xy* 3D-DNA-PAINT images of gp24. C) *xz* 3D-DNA-PAINT images of gp24. Scale bar are 100 nm.


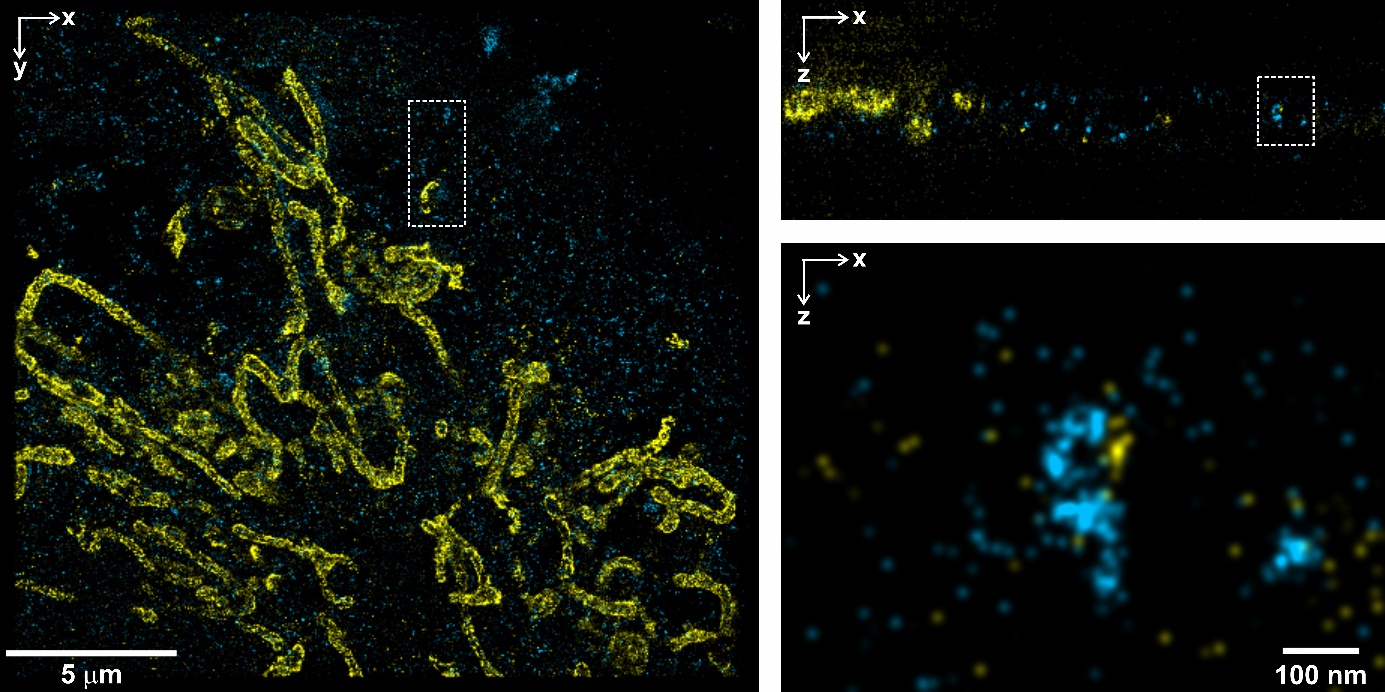


Figure S13: Exchange 3D-DNA-PAINT image showing COS-7 cell mitochondria (yellow) and T4 virus (blue). The right panel corresponds to the highlighted *xz*
cross-section of the zoomed-in area.

Video S1-S2: T4 particles 3D representation.

Video S3: T4 head average particle.
